# The functional logic of corticostriatal connections

**DOI:** 10.1007/s00429-016-1250-9

**Published:** 2016-07-13

**Authors:** Stewart Shipp

**Affiliations:** 1 Department of Visual Neuroscience, UCL Institute of Ophthalmology, Bath Street, London, EC1V 9EL UK; 2 Stem Cell and Brain Research Institute, INSERM U1208, 18 Avenue Doyen Lépine, 69675 Bron, France

**Keywords:** Basal ganglia, Caudate, Putamen, Corticostriatal, Intratelencephalic, Indirect pathway, Action value, Medium spiny projection neuron, Bridging collaterals

## Abstract

Unidirectional connections from the cortex to the matrix of the corpus striatum initiate the cortico-basal ganglia (BG)-thalamocortical loop, thought to be important in momentary action selection and in longer-term fine tuning of behavioural repertoire; a discrete set of striatal compartments, striosomes, has the complementary role of registering or anticipating reward that shapes corticostriatal plasticity. Re-entrant signals traversing the cortico-BG loop impact predominantly frontal cortices, conveyed through topographically ordered output channels; by contrast, striatal input signals originate from a far broader span of cortex, and are far more divergent in their termination. The term ‘disclosed loop’ is introduced to describe this organisation: a closed circuit that is open to outside influence at the initial stage of cortical input. The closed circuit component of corticostriatal afferents is newly dubbed ‘operative’, as it is proposed to establish the bid for action selection on the part of an incipient cortical action plan; the broader set of converging corticostriatal afferents is described as contextual. A corollary of this proposal is that every unit of the striatal volume, including the long, C-shaped tail of the caudate nucleus, should receive a mandatory component of operative input, and hence include at least one area of BG-recipient cortex amongst the sources of its corticostriatal afferents. Individual operative afferents contact twin classes of GABAergic striatal projection neuron (SPN), distinguished by their neurochemical character, and onward circuitry. This is the basis of the classic direct and indirect pathway model of the cortico-BG loop. Each pathway utilises a serial chain of inhibition, with two such links, or three, providing positive and negative feedback, respectively. Operative co-activation of direct and indirect SPNs is, therefore, pictured to simultaneously promote action, and to restrain it. The balance of this rival activity is determined by the contextual inputs, which summarise the external and internal sensory environment, and the state of ongoing behavioural priorities. Notably, the distributed sources of contextual convergence upon a striatal locus mirror the transcortical network harnessed by the origin of the operative input to that locus, thereby capturing a similar set of contingencies relevant to determining action. The disclosed loop formulation of corticostriatal and subsequent BG loop circuitry, as advanced here, refines the operating rationale of the classic model and allows the integration of more recent anatomical and physiological data, some of which can appear at variance with the classic model. Equally, it provides a lucid functional context for continuing cellular studies of SPN biophysics and mechanisms of synaptic plasticity.

## Introduction

**Fig. 1:**
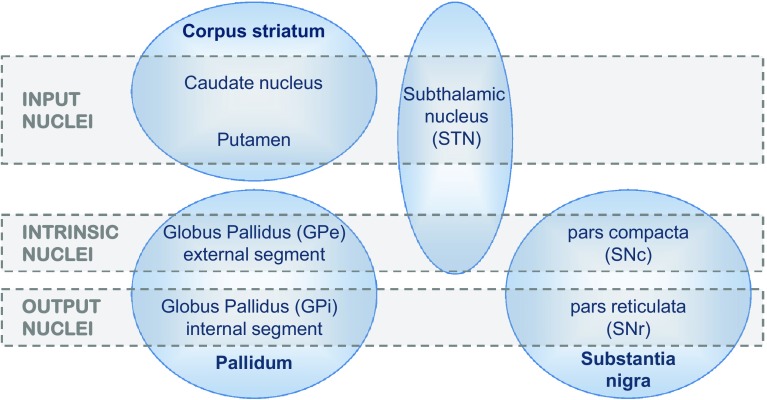
Components of the basal ganglia. The diagram distinguishes the anatomical identity of nuclei (shown in *blue ovals*) from their functional role, as assessed by input/output connectivity (indicated by *grey bands*). The corpus striatum can be considered a single nucleus, perforated by the internal capsule, and named for the strands of grey matter that stretch between the caudate and putamen. The caudate is typically referred to as a ‘nucleus’ whilst the putamen is not, though their cellular constitution is much the same. These two subdivisions are also known collectively as the dorsal striatum, as opposed to the ventral striatum which incorporates the nucleus accumbens (not shown here). Similarly, the two components of the output module, the substantia nigra pars reticulata (SNr), and the internal segment of the globus pallidus (GPi) also share a similar cellular composition and a continuous connectional topography, despite being quite separate anatomically. The subthalamic nucleus (STN) combines both extrinsic (cortical) and intrinsic inputs—the latter originating from another intrinsic nucleus, the external segment of the globus pallidus (GPe). Finally, the substantia nigra pars compacta (SNc) has reciprocal connections with the striatum, though it also receives extrinsic inputs. The striatum, GPe, GPi and SNr all comprise GABAergic projection neurons; the striatum also has several types of identified interneurons, one cholinergic plus three GABAergic. The STN is the only glutamatergic nucleus, comprising just one cell type. The SNc has dopaminergic projection neurons that issue collaterals to several BG nuclei in addition to their main target, the striatum

‘Basal ganglia’ is the accepted collective term for a set of structures in the basal forebrain, now known to form several parallel feedback loops with frontal cortex. In functional terms, there are just five principal components to the basal ganglia (BG), but they enjoy a rather richer anatomical lexicon, whose mastery is the initial hurdle to a deeper appreciation of their fascinating inter-relationships. Take but one example: the substantia nigra and the globus pallidus may be named for their contrasting dark and pale appearance, respectively, yet one BG component—its output module—is an amalgam of sub-parts from each. Figure 1 clarifies all such terminological issues. The simplest conception of the BG loop is that the principal module receiving cortical input, the striatum, directly feeds the BG output module, that communicates back to the cortex via the thalamus. As the initial corticostriatal input is non-reciprocal, the loop as a whole is unidirectional, despite the presence of retro-connections at some stages (e.g. pallidostriatal, corticothalamic). The presence of additional, intrinsic BG nuclei provides for a variety of alternative loops through the system that are set out below.

There is no single concept that adequately captures all known aspects of BG functionality. The proposal that the BG play a role in action selection comes closest to this ideal, especially if ‘action’ is extended to include cognitive events and emotional states, with the implicit idea that the BG act upon prefrontal, limbic and motor cortex in analogous fashion (Mink 1996; Redgrave et al. 1999; Frank 2011). The other principal functional dimension is learning, from simple habit formation to complex motor sequences (Graybiel 1995; Balleine et al. 2009; Jin and Costa 2015). Together these processes can be said to optimise behavioural repertoire in pursuit of reward. The underlying neural plasticity hinges upon phasic dopamine release, signalling reward or its expectation (Montague et al. 1996; Schultz 1998, 2013), and acting mainly within the striatum of the BG to enhance or depress synaptic strength (Centonze et al. 2001; Reynolds and Wickens 2002).

This article will begin an analysis of BG function with a focus upon corticostriatal anatomy; it continues an occasional ‘Functional Logic’ series, aiming to discern functional principles by characterising the structure and organisation of neural circuits (Zeki and Shipp 1988; Shipp 2003). For the basal ganglia this is a challenging synthesis indeed, given the accumulated density of research and the multiplicity of functional dimensions it has uncovered. But there are also well-thumbed blueprints of BG circuitry and models of its operation on which to build. These are presented in the following section, preceded by a brief sketch to help outline the division of labour between the present article and subsequent instalments.

### In a nutshell…

If the BG participates in action selection, this is not to register all the attendant details of the action or how it should be executed. The BG circuitry need only receive a token representation sufficient to indicate that the action in question has entered a state of planning. The purpose of the BG circuit is to evaluate its reward earning potential with respect to alternative actions contingent upon all relevant factors; these factors constitute the context of the action and include interpretations of the sensory environment, internal states, and the planning status of other actions, either complementary or alternative. We can refer to this token as a ‘bid’ lodged by a functional subunit of frontal cortex, whose salience reflects the evaluated context, and which competes with the rival bids to traverse the BG circuit and bias cortical selection in favour of its parent plan.

This, first stage of enquiry is to examine corticostriatal function: to consider how signals conveying a bid for action selection or its context are distinguished, how context separates into positive and negative reward contingencies and how these may be evaluated. A subsequent stage will focus upon neural mechanisms of plasticity, exploiting the oculomotor physiology of certain tasks, such as the antisaccade paradigm, where the reward status of a specified motor action can be arbitrarily manipulated by instructional cues. Up to this point much of the discussion will centre on the striatum, which is where the plastic combinatorial encodings and the schism into ‘good’ and ‘bad’ is thought to take place. The concluding stage will better analyse the nature of competition between bids throughout the BG circuit, as they vie to complete the loop and confer a selective advantage on the cortical representation of the planned action. Throughout, the primary source of reference will be the primate BG system. Material from the rodent BG will be drafted in where it is more informative,^1^ but not to present a comparative analysis per se.

## Founding conceptions of the cortico-BG loop

### Classic models

**Fig. 2:**
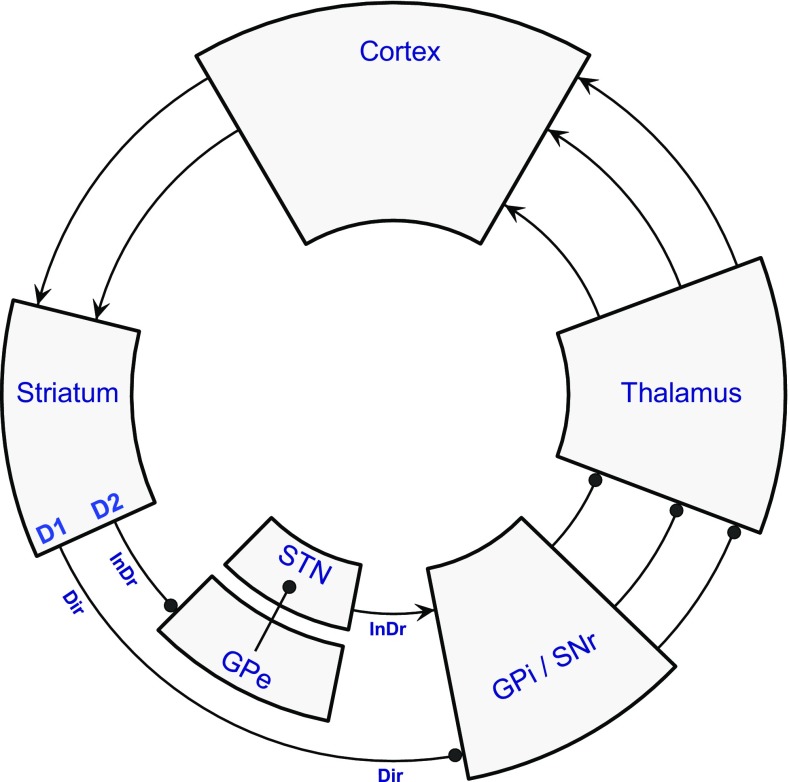
The classic direct/indirect pathway model of BG circuits. This diagram is adapted from the circuit diagrams originally presented by Albin et al. (1989) and DeLong (1990) showing circuit elements common to both that form the essential components of the direct and indirect BG loops with cortex. Operationally, these two loops may be said to traverse the whole circuit, but they are only anatomically distinct in the sector of the loop between the striatum and the GPi/SNr. The direct pathway originates from GABAergic striatal spiny projection neurons (dSPNs) that express D1 dopamine receptors, and project directly to either component of the GABAergic BG output module, GPi/SNr. These two successive inhibitory relays (striatum and GPi/SNr) can achieve positive feedback to the cortex through disinhibition of the thalamus. The classic indirect pathway originates from iSPNs that express D2 dopamine receptors, and project to the BG intrinsic nucleus, GPe; it then passes to the GPi/SNr via the glutamatergic intrinsic nucleus, the STN. Hence, the indirect pathway is pictured to disinhibit the STN, excite the GPi/SNr and achieve negative feedback to the cortex through suppression of the thalamus. Note that a subsequently discovered projection from GPe to GPi/SNr (see Fig. 3) provided a shorter, but logically equivalent route for the indirect pathway, prompting more sophisticated functional models. *Arrowhead ending* excitatory connection, *ball ending* inhibitory connection, *Dir* direct pathway, *InDr* indirect pathway. See Fig. 1 for BG nuclei abbreviations

**Fig. 3:**
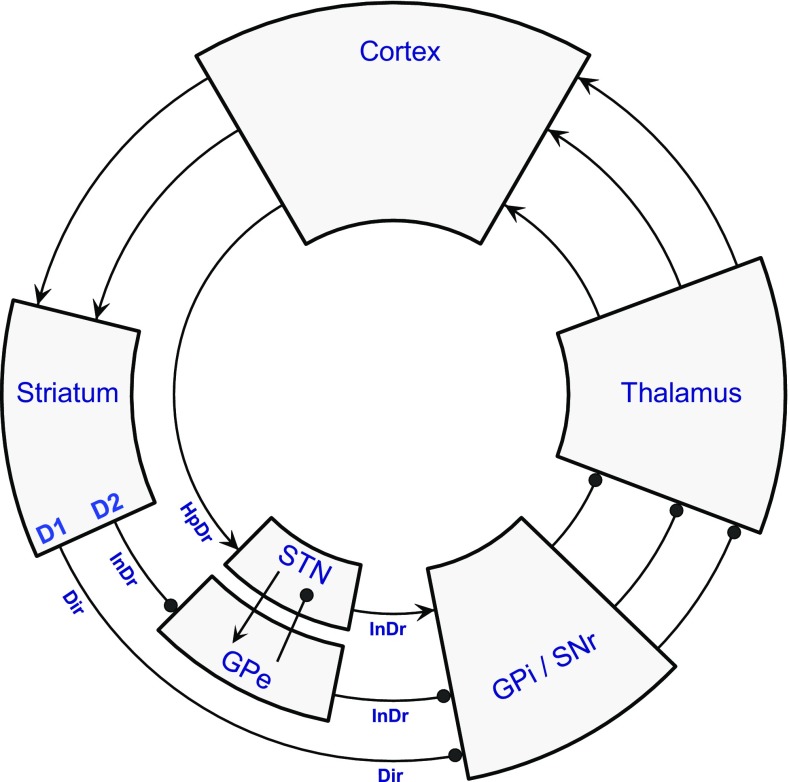
The classic model with added circuit elements. This extended version of the direct/indirect pathway model was the basis for the first generation of computational/neural network models of BG circuit function. It has three additional circuit elements: (1) Direct inhibitory output from GPe to GPi/SNr has a negative effect upon GPi/SNr activity, as does the longer route, via STN, so this was accounted a second limb of the indirect pathway; both routes cause an enhancement of GPi/SNr activity following inhibitory input to GPe from striatal iSPNs. (2) Excitatory cortical input to the STN transmits an excitatory influence to GPi/SNr, and this disynaptic route from cortex to the BG output module was termed the ‘hyperdirect pathway’ (HpDr). As cortically driven activity in the indirect pathway causes disinhibition in STN, the hyperdirect and indirect pathways both exert a positive influence upon STN activity. (3) The STN output is directed to both components of the globus pallidus; hence, the GPe and STN are reciprocally connected, potentially giving rise to oscillatory dynamics. Conventions as for Fig. 2

The original circuit models aimed to rationalise how BG lesions or degenerative conditions could give rise to either hyperkinetic or hypokinetic motor symptoms (Albin et al. 1989; DeLong 1990). The key lay in the identification of two separate classes of striatal spiny projection neuron (SPN), with distinct patterns of projection and neurochemistry, if alike in cellular morphology. Figure 2 formulates the resulting pair of parallel loop circuits through the BG nuclei. A unique feature of these circuits is serial connectivity through inhibitory projections. The so-called ‘direct’ pathway has two such links and the ‘indirect’ pathway has three, such that the two loops effect positive and negative feedback, respectively. The striatum forms the initial inhibitory step; it receives excitatory cortical input, but the striatal SPNs are GABAergic with low spontaneous activity. Subsequent GABAergic nuclei in the pathways (the external and internal components of the globus pallidus, GPe and GPi, and the substantia nigra pars reticulata, SNr) have high tonic firing rates, such that excitatory influences can be conveyed via disinhibition of their respective target regions (Chevalier and Deniau 1990). Thus, striatal output from direct pathway SPNs (dSPNs) inhibits the BG output module, GPi/SNr, causing disinhibition of the thalamus; conversely, striatal output from indirect pathway SPNs (iSPNs) inhibits the BG intrinsic nucleus, GPe, ultimately causing the reverse effect upon the thalamus, enhanced inhibition (see Figs. 2 and 3 for details).

Apart from their opposing actions, a second key feature of the direct and indirect pathways is their differential regulation by dopamine (Albin et al. 1989; Gerfen and Surmeier 2011). The source of dopaminergic input to the striatum is the substantia nigra pars compacta (SNc), which is fed by a reciprocal input from the striatum but also by external sources, and acts as a modulatory gateway to BG circuits (Schultz 1998). In addition to mediating long term plasticity, noted above, dopamine also has a short-term influence upon striatal activity; it enhances the excitability of dSPNs and has the opposite effect upon iSPNs. It is this property that gave a fundamental insight into the pathogenesis of contrasting motor disturbances; for example, depletion of dopamine resulting from nigrostriatal degeneration in Parkinson’s disease could cause hypokinetic symptoms by augmenting negative feedback to the motor cortex from the indirect pathway, and attenuating positive feedback delivered by the direct pathway. Conversely, hyperkinetic conditions could be attributed to impairment of the indirect pathway; for instance, selective degeneration of iSPNs (at least at the initial stage) of Huntington’s disease, causing an inability to suppress involuntary movements (Albin et al. 1989; DeLong 1990).

A second form of parallelism in BG circuits concerns the maintenance of cortical topography through the loop. A striking feature of gross BG anatomy is profound convergence, signified by the contraction in tissue volume as the pathways proceed from cortex to striatum and thence to the pallidum and nigra, and the progressive reduction in neuron numbers at each step (Oorschot 1996; Hardman et al. 2002); notably, the putamen and globus pallidus are so-shaped in transverse sections as to merit a picturesque corporate term, the ‘lentiform’ (lens-like) nucleus. The traditional interpretation of this macroscopic funnelling was that it indicated some kind of loss of identity—an integration of cortical influences, or perhaps even a competition as to which might survive the bottleneck. However, tract-tracing studies later identified discrete regions of frontal cortex each of which, to a first approximation, actually maintains its territory throughout the BG loop such that the re-entrant projection from the thalamus returns to its original cortical source (Alexander et al. 1986; Alexander and Crutcher 1990). This is termed a ‘closed loop’, a configuration that is not incompatible with the local existence of direct and indirect pathways looping through each node in the topography. The organisation is also held to extend to finer levels; for example, the basic somatotopy of motor cortex is maintained throughout subsequent stations in both these BG loops (Romanelli et al. 2005; Nambu 2011). This principle, originating with the classic BG models—the existence of ‘microchannels’—has since been near universally adopted by neural network models of BG function.

### Neural network modelling of BG functional mechanisms

Network models,^2^ using diverse strategies to compute neural function and interaction, clarify the dual forces opposing a bid for action selection; competition from rival bids seeking to access the direct pathway, and cancellation by the indirect pathway (Schroll and Hamker 2013). To do so, they commonly incorporate three additional circuit elements (shown in Fig. 3), plus some details of microcircuitry. The first addition is a second, shorter limb of the indirect pathway. The indirect pathway was originally designated to pass from the GPe to the BG output module via the excitatory subthalamic nucleus (STN)—a non sign-reversing relay as the STN comprises exclusively glutamatergic projection neurons. The added component is formed by collateral axons of the GPe projection to STN that terminate in either or both nuclei of the output module, GPi and SNr (Smith et al. 1998; Sato et al. 2000a). Logically, each limb of the indirect pathway has a similar, positive effect upon BG output and consequent thalamic suppression; following cortical activation of striatal iSPNs and inhibition of GPe activity, the short limb causes disinhibition of the output module GPi/SNr and the long limb disinhibits the STN, enhancing its excitatory output to GPi/SNr. The two routes for the indirect pathway are known to converge at the single neuron level within GPi/SNr, although they are far from equivalent, since GPe axons terminate more focally than STN axons and with a more proximal distribution of dendritic contacts; there is actually a 3-way convergence, as direct pathway terminals from striatal dSPNs also contact the same GPi/SNr output neurons (Parent and Hazrati 1995b; Smith et al. 1998).

The second and third additional circuit elements are connections of the STN: its receipt of excitatory input from motor (and prefrontal) cortex, and its output to GPe (formed by collaterals of axons terminating in GPi/SNr) (Parent and Hazrati 1995b; Sato et al. 2000b). The cortical influence upon the STN is concordant with the disinhibitory influence of the long limb of the indirect pathway. Both effects can oppose action selection with the STN exciting the BG output nuclei and hence inhibiting the return thalamocortical pathway. The cortical influence upon STN is the more immediate, and because this establishes another negative feedback loop to the cortex (one with a single inhibitory step) it was termed the ‘hyperdirect’ pathway—by analogy to the classic model—and proposed to act as a short-term restraint upon voluntary movement (Mink 1996; Nambu et al. 2002b). However, the fact that the STN innervates GPe in addition to GPi/SNr complicates the picture; a number of interactions become possible, as mentioned below.

**Fig. 4:**
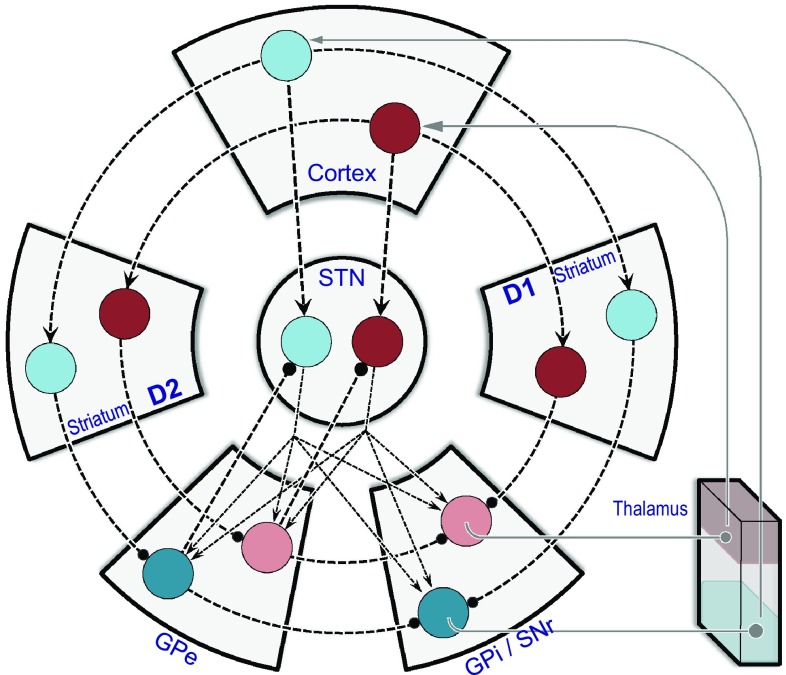
Architecture of a computational BG network model. The circuit diagram shows two microchannels, indicated by connections amongst two sets of *blue* or *red discs*, specific for two alternative actions (the actual computational implementation of the model used six microchannels). The format is similar to Figs. 2 and 3, with some adjustment to accommodate the additional wiring. For instance, D1 (dSPN) and D2 (iSPN) components of the striatum are here represented by separate blocks. Each disc denotes a population of neurons, modelled by its normalized mean firing rate (*dark* for highly active, *pale* for less active). The ‘*red*’ action is the one selected by the model in the state illustrated. Note that most connections are channel specific (1 disc: 1 disc); these include the graphically circular pathways between cortex, striatum, GPe and GPi/SNR, as well as both sets of inputs to the STN at the centre (from cortex, and from GPe). Competition between the ‘*blue*’ action and the ‘*red*’ action is mediated by the outputs from the STN that are one-to-many (1:2 in the diagram; 1:6 in the computational implementation). This representation is adapted from Gurney et al. (2015), but the network architecture is equivalent to earlier implementations of the model (Gurney et al. 2001a, b; Humphries et al. 2006). Outputs from GPi/SNr to thalamus and from thalamus to cortex are shown for completeness; thalamic activity was not part of the model. Conventions as for Fig. 2

As remarked above, all network models invoke a sample set of microchannels, each of which constitutes serial focal connections from station to station through various BG loops. However, some stages utilise diffuse connectivity, in which each microchannel connects with all others—typically the output from STN to GPi/SNr. So, for example, in the context of motor circuitry, a bid for action selection is implemented by a direct pathway input to GPi/SNr, and opposed by the background activity of all rival bids, mediated via the STN. Hence, in this setup, competition between bids is enacted by opponency between the direct and hyperdirect pathways (Gurney et al. 2001a, b; Frank 2006; Humphries et al. 2006; Leblois et al. 2006; Wiecki and Frank 2013); see Fig. 4 for an example model architecture.

There is then the question of the relative roles played by the long and short limbs of the indirect pathway. The simplest view of the former is that disinhibition of STN via GPe can mimic the restraining action of the hyperdirect pathway (Mink 1996; Wei et al. 2015). An alternative proposition is that reciprocal connections between STN and GPe form a negative feedback loop, acting to quash the initial cortical excitation of STN, and thus *terminating* the restraint on movement imposed by the hyperdirect pathway (Frank 2006; Wiecki and Frank 2013). Another model family attributes this reciprocal circuitry with the role of ‘capacity scaling,’ adjusting the level of hyperdirect restraint to afford selection of one bid against variable levels of competition (Gurney et al. 2001a, b; Humphries et al. 2006). The short limb of the indirect pathway passing straight from GPe to GPi/SNr is typically allotted focal connectivity, befitting a specific role of bid cancellation. In several models, it is proposed to carry a learnt ‘stop’ (or ‘no-go’) signal, embodying the negative context of a bid; these studies aim to model plasticity over a course of trials in which loss of reward progressively strengthens the stop signal, finally outweighing the direct pathway where the two converge at the GPi/SNr stage of their conjoint microchannel (Brown et al. 2004; Frank 2005, 2006; Baladron and Hamker 2015). Other models are more radical in their treatment of the indirect pathway, citing concerns that the direct and indirect pathways are not nearly as distinct as the classical scheme portrays. One, for example, essentially cuts it out of the model architecture altogether (Leblois et al. 2006). Another—sporting a highly sophisticated anatomical and physiological specification—allots the short indirect pathway connection from GPe to GPi a diffuse organisation (i.e. a one-to-many communication across channels), essentially replacing the hyperdirect pathway as a source of restraint, the latter being locked to baseline levels of activity (Lienard and Girard 2014). A final variation (Brown et al. 2004) notes that the cortical input to the STN is formed exclusively by collaterals of executive cells in layer 5B (and not by ‘planning’ corticostriatal cells in other layers)^3^, and from that perspective is not suited to a role as the initial source of restraint. Hence, the hyperdirect pathway is engaged at a later stage—its role is to lock out rival bids whilst the selected bid is executing. Competition between bids in this model is achieved by a different mechanism, namely feedforward inhibition between rival bids at the level of the striatum, mediated through corticostriatal inputs to a population of fast-spiking interneurons directly contacting dSPNs (Brown et al. 2004).

If nothing else, it is plain from this short survey that BG network models explore a number of variant functional architectures that are not fully constrained by the available anatomical evidence. Thus specific issues, such as the reliability of the distinction between the classic direct and indirect pathways, and laminar variations in the functionality of corticostriatal output neurons, are worth exploring in more detail. Beyond that there is yet more circuitry to consider—a number of ‘shortcuts’, subcortical loops formed by BG nuclei, brainstem structures and thalamus, whose functional contribution remains uncertain: see Box 1 for a summary. Capping it all, however, there is a crucial dimension of cortico-BG function that has escaped modelling altogether, and this is the means by which the striatum fashions the salience of a bid, according to the momentary context. Salience in the above models is adjusted by the operator; it does not evolve from considerations of corticostriatal anatomy. As will be seen, the neural mechanism of context evaluation heavily depends upon the very particular physiology of SPNs, but how (or if) an input representing context is processed differently from an input conveying a bid for action selection is little known, and rarely considered. The first step is to consider the anatomical basis of the closed-loop organisation, since this is the justification for the modelled microchannels, and because the very nature of ‘context’ implies that a closed loop should not function in isolation.

## Topographic organisation of the cortico-BG loop

### Open and closed loops

The strict notion of the ‘closed loop’, introduced above, implies a private channel of communication that neither receives nor transmits any influence upon neighbouring channels. Alexander et al. (1986) originally identified five closed circuits: motor, oculomotor, lateral prefrontal, medial prefrontal and limbic. Whilst the network modellers’ microchannel extends this principle to the level of representation of single actions (at least within motor circuits) the subsequent trend of topographic anatomy has moved in the opposite direction, with the number of principal BG domains reduced to just three: sensorimotor, cognitive/prefrontal and affective/limbic (Parent and Hazrati 1995a; Joel and Weiner 2000; Postuma and Dagher 2006; Haber and Calzavara 2009; Sadikot and Rymar 2009). These represent, naturally enough, the three major functional subdivisions of frontal cortex. The input to BG circuits, however, derives from all four cortical lobes; in fact, there are striatal projections from virtually the entire cortical sheet, bar area V1.^4^ Much of the input from the occipital and temporal lobes, in particular, is directed to the long C-shaped tail of the caudate nucleus, as it wraps around the lateral ventricle. But all of this input is integrated within BG circuitry and returned to frontal cortex, amounting to an open-loop input architecture. As will be seen, the precise patterning of corticostriatal inputs is complex and multidimensional and, as a prelude, may be contrasted with the more focal and conceptually simpler organisation of the return component of the loop.

### Discrete BG output channels in the return loop to cortex

As a generalisation, a closed-loop architecture is more characteristic of the corticopetal sector of the BG loop than the corticofugal. The technical demands of determining precisely what connects with what through successive BG stations are tricky, and the most satisfactory method is the use of neurotropic viruses, such as herpes and rabies, to achieve trans-synaptic retrograde transport (Hoover and Strick 1993; Dum and Strick 2013). The uniform study design to date has been to place virus at strategic cortical sites, to observe disynaptic labelling (via thalamus) within the BG output nuclei, and trisynaptic labelling of the STN, GPe and striatum. The subnuclear location of the viral-labelled neurons has been found to depend on the exact site of virus deposition within cortex, and comparison across cases allows inference of cortical topography within each BG nucleus.^5^

The aggregate of this work indicates a topographic map of motor and prefrontal cortex extending across the two BG output nuclei, GPi and SNr (Middleton and Strick 2000). The precision in this arrangement has justified the initial description of discrete ‘output channels’ (Hoover and Strick 1993). Studies typically indicate a local gradient within the GPi and SNr, reflecting the relative locations of cortical sites (Hoover and Strick 1993; Middleton and Strick 2002; Akkal et al. 2007; Saga et al. 2011). For motor cortex, of course, this implies a somatotopic representation, as confirmed for M1 (Hoover and Strick 1999); but each motor area—M1, PMv and SMA—is associated with a distinct somatotopic map, as three separate foci of viral-labelled neurons are found if corresponding (forelimb) sites are selected for injection of tracer in each area (Hoover and Strick 1993). These somatotopic maps are mainly within GPi, except for the orofacial representation of the M1 map, that extends from GPi to the adjacent region of SNr (Hoover and Strick 1999)—indicating that the two output nuclei, GPi and SNr, may form a single, conjoint representation of prefrontal and motor cortical territory. The SNr is the exclusive source of relays to ventral prefrontal cortex (areas 46v and 12) (Middleton and Strick 2002), including the caudolateral margin of the SNr that communicates with the frontal eye field (FEF) (Lynch et al. 1994). Likewise, the GPi dominates medial premotor cortex (areas F3/SMA and F6/pre-SMA) (Akkal et al. 2007) but there is a broad crossover region of dorsal premotor and prefrontal cortex where areas such as F2/PMd and 9 receive relays from both GPi and SNr (Middleton and Strick 2002; Saga et al. 2011).

Notably, the viral methodology has certified two sites *outside* the frontal lobe that also receive BG relays from SNr; these are areas TE (Middleton and Strick 1996) and AIP (Clower et al. 2005), situated in inferotemporal and parietal cortex, respectively. There may be others too, as the list of post-rolandic cortical areas tested in this way is not extensive. This observation evidently qualifies the nature of the cortical output map across the SNr, as TEO and AIP are far from adjacent to prefrontal cortex. The topography within SNr (and GPi) may thus be characterised by some form of dislocation, and has yet to be exhaustively mapped; so far, it does not show duplication (i.e. twin foci within one nucleus relaying to a single site in cortex), nor give any sign that a single locus within the BG output nuclei may communicate with multiple sites in cortex. In this respect, it satisfies the precepts of closed-loop circuitry.

The same conclusion is less immediate when considering trisynaptic labelling, e.g. as seen in the striatum, stepping one stage back in the direct pathway. Somatotopic trends are still evinced by viral injections at different sites in M1 (e.g. hindlimb, proximal and distal forelimb, and orofacial), but the clusters of viral-labelled neurons are less focal, and more interspersed (Miyachi et al. 2006). One study compared nearby viral injections in rostral and caudal sectors of dorsal premotor area F2, and describes neurons projecting multisynaptically to F2r or F2c as being ‘intermingled’ across a broad territory in the striatum, in contrast to the notably more segregated distribution observed in GPi and SNr (Saga et al. 2011). Overlapping distributions were similarly inferred in GPe and STN (i.e. trisynaptic labelling in the indirect pathway) suggesting a similar erosion of topographic organisation (Saga et al. 2011). The closed-loop formulation can still apply here, depending upon two provisions. One, most obviously, is that the neurons projecting multisynaptically back to M1 (or F2) fall within the territory innervated by corticostriatal afferents from M1 (or F2); this is true for the main proportion of viral-labelled neurons that occur within the dorsal, sensorimotor part of the striatum (Kelly and Strick 2004; Miyachi et al. 2006; Saga et al. 2011). However, there is typically also a second group, occurring more ventrally in limbic striatum, well-removed from the motor corticostriatal afferents; as such, this group is said to form an ‘open-loop’ circuit (Kelly and Strick 2004; Miyachi et al. 2006; Saga et al. 2011). The second provision is that individual SPNs do not contribute to more than one output channel. This remains uncertain for cases of intermingling, such as F2c and F2r noted above, given the limitations of viral technology (see footnote 5). For other examples, such as M1 vs. prefrontal area 46, the respective distributions of trisynaptic rabies-labelled cells are well separated across the striatum, consistent with closed-loop circuitry (Kelly and Strick 2004).

The indication is that the discrete BG output channels are not directly inherited, as such, from strict topographic order in the corticostriatal pathway but are synthesised, at least in part, by topological reordering within the cortico-BG loop. Such an organisation follows what is known as the ‘divergence–reconvergence’ strategy for trans-striatal circuitry, originally coined to describe connections from a single somatotopic locus in M1 (or S1) to a single corresponding locus in GPi that were shown to relay through multiple segregated patches of the striatum (Flaherty and Graybiel 1994). To consider that in more detail, we switch to the anatomical fulcrum of the matter, an examination of BG input topography at source.

### Topographic organisation of corticostriatal afferents

The corpus striatum is named for the striations formed by the cellular bridges linking the caudate and putamen across the internal capsule. Though anatomically separate, these two nuclei are best considered a single functional entity. A more meaningful subdivision of striatal territory is the distinction between striosomes and matrix (Graybiel 1990; Crittenden and Graybiel 2011). The former, appearing as lighter patches in histological sections stained for acetylcholinesterase activity, occupy about 20 % of the striatal territory. Striosomes are distinct in multiple neurochemical attributes, connectivity, and in shaping dendritic fields that often respect compartment boundaries. The striosome compartment mediates control of dopaminergic reward mechanisms and is integral to limbic BG circuitry, receiving convergent input from orbitofrontal, cingulate and insular cortex (Crittenden and Graybiel 2011; Fujiyama et al. 2015). It is the matrix compartment of the striatum, serving the remainder of the cortex, upon which the examination of corticostriatal topography will focus.

The original concept of corticostriatal mapping was a simple topological transformation of the cortical mantle, albeit respecting the obvious constraints imposed by rendering such a map within the complex three-dimensional volume of the striatum (Kemp and Powell 1970). Even so, the functional interpretation emphasised integration, noting substantial overlap in all dimensions between adjacent projection zones such that no part of the striatum was likely to fall under the sole influence of one functional area of cortex (Kemp and Powell 1970, 1971). The original report of head-to-toe somatotopy, expressed by M1 projections along a ventro-dorsal gradient in the putamen, also referred to the likelihood of overlap between head and arm, and arm and leg territories (Kunzle 1975). Visual cortex is relatively underrepresented with V1 absent, as noted above, and V2 making meagre connections to the ‘genu’ of the caudate tail (Saint-Cyr et al. 1990). Much of the concentric belt of occipito-temporal visual cortex also projects mainly to the nearest component of the caudate and/or putamen, conforming to the concept of a simple, if somewhat diffuse topography (Saint-Cyr et al. 1990).

The global topographic concept ran into problems with the demonstration of longer range forms of overlap. For example, frontal and parietotemporal regions of cortex both showed a longitudinally extended zone of projection, each invading the other’s topographic heartland (Yeterian and Van Hoesen 1978; Van Hoesen et al. 1981; Selemon and Goldman-Rakic 1985). Furthermore, there was a ‘mosaic’ quality of organisation, in that projections from a single area in cortex were not only locally patchy, but also discontinuously distributed to separate striatal sectors, e.g. frontal projections to head, body and tail of the caudate nucleus (Yeterian and Van Hoesen 1978). Diagnosing some regularity in the gathering complexity, Yeterian and Van Hoesen (1978) proposed this generalisation: that areas with directly reciprocal corticocortical connections appeared to project, at least in part, to the same sectors of the striatum [hereafter termed the ‘YVH’ principle]. Several pairs of cases were examined to demonstrate the reliability of this principle, and its obverse, that non-connected areas would fail to share common zones of striatal projection (Yeterian and Van Hoesen 1978).

To establish precise coincidence of corticostriatal projections from separate cortical origins, it is necessary to avoid comparison across cases by employing dual-tracer techniques. The first purposeful study of this nature immediately arrived at a different conclusion, emphasising interdigitation rather than superimposition of patchy projections from interconnected cortical areas (Selemon and Goldman-Rakic 1985). Subsequent work has, however, affirmed the general veracity of the YVH principle. For instance, projections from the two interconnected oculomotor regions of frontal cortex, the frontal and supplementary eye fields (FEF and SEF) were found to coincide on a patch-for-patch basis within the region of overlap of the two fields (Parthasarathy et al. 1992). And demonstrating the obverse of the YVH principle, no coincidence was seen if projections were traced from one eye field and the skeletomotor cortex adjacent to the other eye field (Parthasarathy et al. 1992). In a similar vein, there is also precise, patch-for-patch corticostriatal convergence from the somatosensory area S1 (comprising Brodmann areas 3A, 3B, 1 and 2). Projections from corresponding loci in the somatic maps of these areas terminate in near identical sets of patches in the putamen (Flaherty and Graybiel 1991); these same patches also receive input from corresponding body-loci in M1, although the coincidence is less precise in that patches created by injections of tracer in M1 tend to be significantly larger (Flaherty and Graybiel 1993b, 1995).

**Fig. 5:**
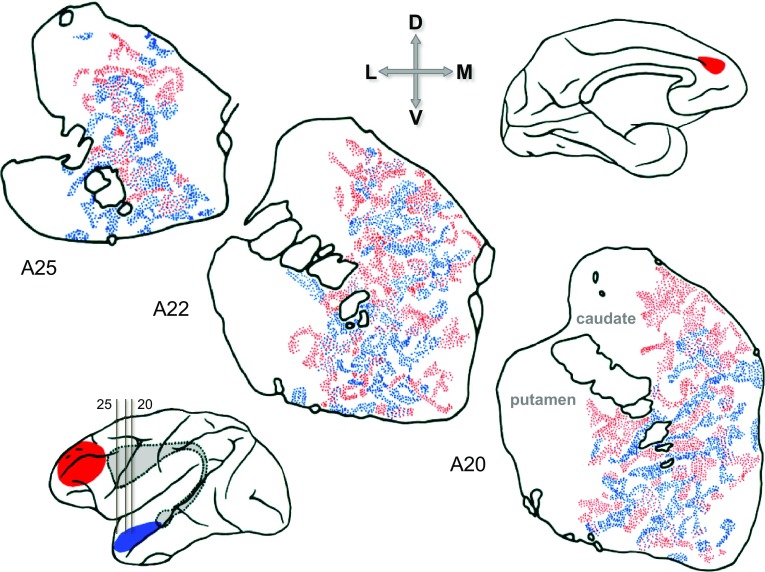
An interdigitating pattern of convergent, patchy corticostriatal terminals. Dual anterograde tracers were placed in anterior temporal cortex (*blue*) and prefrontal-cingulate cortex (*red*). The predominant uptake zone for the ‘*red*’ tracer was located in posterior orbitofrontal and anterior cingulate cortex; importantly, both regions were later shown to be a source of afferents to the striosome compartment of striatum (Eblen and Graybiel 1995). The ‘*blue*’ afferents may be inferred to have invaded the matrix compartment, potentially explaining the predominant interdigitating pattern. Reproduced, with permission of Society for Neuroscience, from Selemon and Goldman-Rakic (1985)

From a later vantage point, the original report casting doubt upon coincident projections (Selemon and Goldman-Rakic 1985) bears some reanalysis. Of the four dual-tracer cases presented, three showed varying extents of overlap,^6^ and roughly proportionate levels of coincidence (i.e. the more overlap between fields of striatal projections, the greater the coincidence between individual patches). These were overshadowed by the fourth, ‘case 18’, that revealed a substantial area of overlap showing almost exclusively interdigitating patches (Fig. 5)—qualitatively a different pattern of organisation and one that, in retrospect, may have reflected segregation between striosome and matrix compartments. The paired placements of tracers for this case were anterior superior temporal, and ‘prefrontal-cingulate’—the latter a consequence of unintended spread of the tracer through frontal white matter into medial cortex. The authors specifically noted (by reference to comparable single tracer cases) that most corticostriatal afferents could be attributed to the orbitofrontal and cingulate components of this large site. Crucially, these very regions of limbic prefrontal cortex were later shown to be a specific source of projections to the striosome compartment (Eblen and Graybiel 1995).

**Fig. 6:**
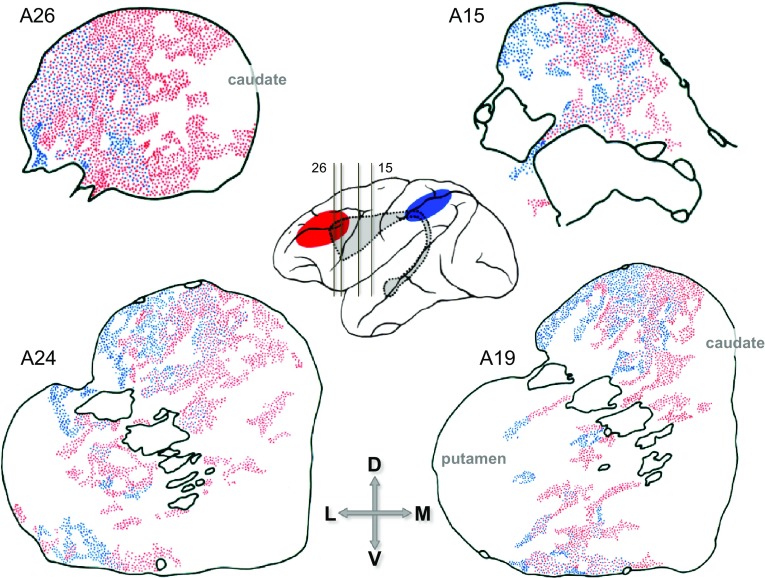
A superimposing pattern of convergent, patchy corticostriatal terminals. Dual anterograde tracers were placed in frontal area 46 (*red*) and parietal area 7 (*blue*). Afferents from both sources invade the matrix compartment and the pattern of local overlap is predominantly (but not exclusively) one of superimposition/coincidence. Reproduced, with permission of Society for Neuroscience, from Selemon and Goldman-Rakic (1985)

Arguably, this single study—or the single case 18—has been misleadingly influential (it is still cited as a counterweight to the YVH principle). There is no comparable evidence that two patchy projection fields showing extensive overlap within the matrix compartment eschew all coincidence in favour of interdigitation, when the source areas are cortically interconnected. A reasonable conjecture is that the degree of corticostriatal convergence depends upon the relative strength of the cortical interconnection—or, perhaps, upon the extent to which the two areas participate in similar cortical networks. There is some evidence for this in the other three cases from this study. For example, ‘case 14’, pairing frontal (area 46) and parietal (area 7) sites of tracer injection, produced heavily overlapping fields of striatal terminals with near exclusive coincidence in the head of the caudate giving way to equal prevalence of coincidence and interdigitation within the zone of overlap more caudally—see Fig. 6. The explicit description of the two fields as “remarkably distinct” might be justified if the prior expectation had been to observe 100 % coincidence. However, the respective cortical networks of areas 7a and 46 are only partially congruent; a recent study allows estimation of their network overlap at 76 % (Markov et al. 2014).^7^

Whilst the evidence considered so far has supported the YVH principle, there are some discordant observations of varying severity. These all concern corticostriatal projections from motor cortex (primary, premotor, supplementary and cingulate motor areas) that have been subjected to the most systematic investigation. Violations of the YVH principle are occasioned by apparent failures of corticostriatal convergence between cortically connected areas—but not vice versa. Box 2 presents the evidence in more detail, noting the provisional nature of several of these assignments. More significantly, it suggests recasting the YVH principle to the effect that ‘convergent striatal connections always derive from areas that are cortically connected’, as opposed to ‘areas that are cortically interconnected always give rise to striatal convergence’. The strategic difference in formulation prompts us to consider which elements of cortical networks may or may not utilise striatal convergence to implement their specific functions.

### A ‘replication principle’ for the striatum?

Dating back over a similar timescale, studies of the connections between (visual) cortex and thalamus revealed a similar principle: that *“if two cortical areas communicate directly, they are likely to have overlapping thalamic fields; if not, their thalamic fields avoid each other”* (Shipp 2003). Because the indirect cortico-thalamocortical links so created tend to mimic direct corticocortical pathways, this was dubbed the ‘replication principle’ (Shipp 2003). At face value, the term ‘replication’ is an inaccurate descriptor of the YVH principle owing to the lack of a back connection from striatum to cortex. But the underlying relationships do appear to be more cogent, in that the groups of cortical areas making convergent projections to thalamus and striatum tend to be highly similar. For example, areas V4, TEO and TE of the ventral visual pathway have overlapping projection fields within both pulvinar (Shipp 2003), and caudate tail (Saint-Cyr et al. 1990); similarly, homologous subsets of medial, orbital and lateral prefrontal areas can be defined by convergent projections upon either the anterior striatum, or the anterior thalamus (Yeterian and Pandya 1991, 1994).

The homology between corticostriatal and corticothalamic convergence is further emphasised by thalamostriatal projections arising from several thalamic relay nuclei that thereby establish an indirect cortico-thalamostriatal pathway. In another variation upon the theme of the YVH principle, analysis of source neuron fields traced retrogradely from the striatum shows that convergent striatal projections arise from a pair of zones in cortex and in thalamus that are themselves known to be interconnected (McFarland and Haber 2000). The organisation of the connections forming this functional triad has best been documented for the thalamic nuclei relaying BG output signals to motor cortex, namely VA and VL. For instance, ‘executive’ motor cortex (such as M1 and caudal premotor areas) communicates with subunits of VL and VA that share a common striatal target zone in dorsal putamen. By contrast, the more rostral premotor areas communicate with thalamic zones that jointly converge upon the dorsolateral caudate (McFarland and Haber 2000, 2001, 2002).

To take account of the above findings, a generalised ‘replication principle’ could be reformulated thus: *patterns of cortical convergence upon subcortical structures tend to replicate each other, and to mirror transcortical patterns of association; areas of cortex that are not directly connected do not directly converge upon subcortical structures*. This incorporates the original sense but encompasses a broader range of brain connectivity. Yet, whilst summarising common observations from the neuroanatomical literature, it should not be taken as a cast-iron ‘law’ so much as an index of the norm. Specific brain systems may conform (or depart) from the replication principle to greater or lesser extents, which then provides a useful tool to dissect their structure–function relationships.

### Systematising corticostriatal convergence

How far and how well can discrete cortical systems, whose elements share convergent striatal projections, be identified and characterised? One proposal, building on the original YVH principle, is that cortical systems align with the level of differentiation of cortical laminar architecture (layer 4 is decreasingly distinct toward the margin of the cortical sheet—also known as allocortex—whilst the deep layers are more prominent). The frontal lobe, in particular, has been partitioned into separate architectonic trends of increasing laminar differentiation, rooted in separate zones of allocortex; a basoventral trend stemming from paleocortex, and a mediodorsal trend stemming from archicortex (Barbas and Pandya 1989). The frontal areas comprising each trend connect with separate territories in the striatum and thalamus, and are also each more cortically interconnected amongst themselves (Yeterian and Pandya 1991). The patterns identified in this set of connections have subsequently been refined (Ferry et al. 2000), and are all in accord with the broader replication principle (as restated above). This systematisation was further extended to incorporate archi- and palaeocortical trend components of the parietal, occipital and temporal lobes (Yeterian and Pandya 1991, 1993, 1995, 1998), and also insular cortex (Chikama et al. 1997).

Such a global operation of the replication principle allows us to resurrect, in modified form, the original concept of a global topography—or what might now be termed a ‘folded topography’. First, this depends upon the tripartite subdivision of the striatum into limbic, prefrontal and motor domains that can be pictured as a limbo-motor or roughly rostro-caudal gradient in the corticostriatal output of frontal cortex (Haber 2003) (this gradient can have medio-lateral, ventro-dorsal and rostro-caudal polarities in standard anatomical planes intersecting the striatum, but owing to the complex configuration of the striatal volume, is not readily encapsulated in a single Cartesian dimension). Second, the ‘fold’ in corticostriatal topography mirrors the symmetrical organization of parieto-frontal transcortical connections about the central sulcus; S1 connects mainly with M1, the sensory association areas of rostral parietal cortex with caudal premotor cortex, and more caudal visuosensory areas with rostral premotor cortex (Darian-Smith et al. 1993; Matelli et al. 1998; Shipp et al. 1998; Geyer et al. 2000; Adams et al. 2013). These generalisations may just describe the centre of gravity of complex connectional fields, but they are tolerably well replicated in the patterns of corticostriatal convergence. The dual-tracer study of patch-for-patch convergence between S1 and M1 in dorsal putamen (Flaherty and Graybiel 1993b), noted previously, provides one direct example and studies of parietostriatal connections cite many others, drawn from comparison across cases—e.g. convergence from areas LIP and FEF upon the body of the caudate, or convergence from posterior parietal cortex and prefrontal area 46 upon the head of the caudate (Cavada and Goldman-Rakic 1991; Yeterian and Pandya 1993).

**Fig. 7:**
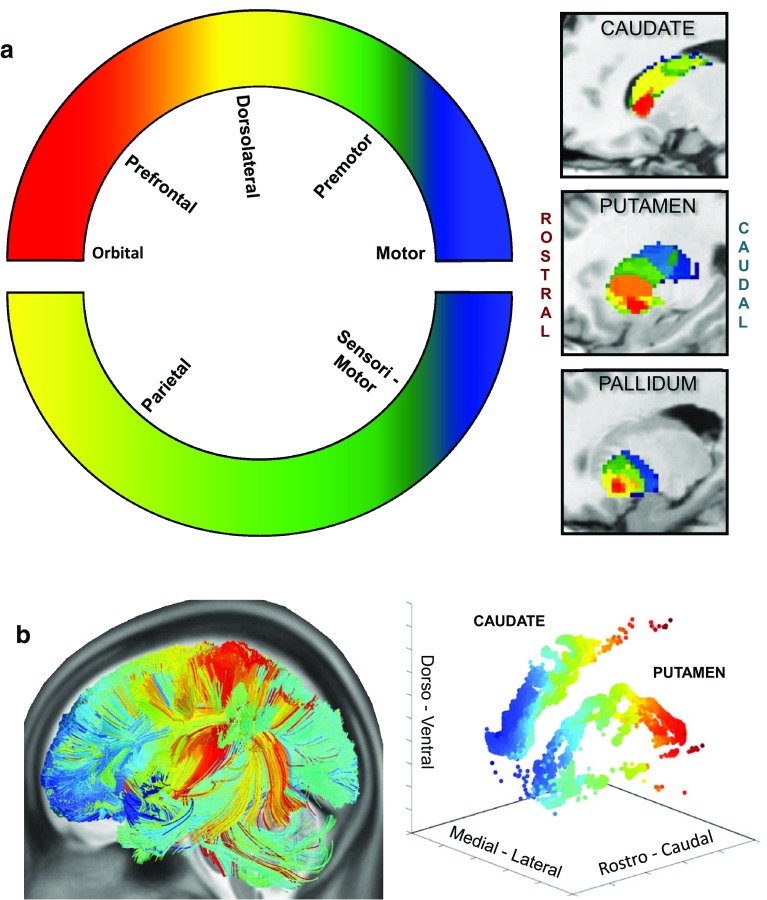
Rostro-caudal connection gradients in human BG nuclei shown by MR diffusion imaging tractography. **a** *Left* a schematic colour map of the origin of corticostriatal fibres (i.e. cortical regions of a given hue project to similarly *colour*-coded striatal and pallidal locations). Note that in the cortical colour map, the rostro-caudal *red*–*blue* colour gradient reverses at the level of motor cortex, i.e. at the junction between the frontal and parietal lobes. At *right*, sagittal sections through caudate, putamen and globus pallidus, showing corticostriatal and cortico-striato-pallidal gradients. Each nucleus shows a monotonic rostro-caudal *red* to *blue* gradient, implying a folded cortical topography about the central sulcus. **b** *Right* a schematic colour map of the termination of corticostriatal fibres (i.e. in this figure the arbitrary colour map is assigned to the striatal volume, not the cortex). Note that the *red–blue* gradient is reversed with respect to **a**: *red* is most caudal. Hence, examining the identified fibre tracks in the cortex, a *deep-blue*–*red* gradient stretches from the frontal pole to the sensorimotor cortex lining the central sulcus. Further caudally, the *yellow*, *pale-green* and mainly *pale-blue* hues of parieto-occipital fibres again signify a folded global topography. **a** Reproduced, with permission of Society for Neuroscience, from Draganski et al. (2008). **b** Reproduced, with permission of Society for Neuroscience, from Jarbo and Verstynen (2015)

Global topographic trends that require multiple, cross-case modelling of corticostriatal connections in monkeys (Averbeck et al. 2014) are more readily discernible using human diffusion imaging tractography (dMRI)^8^ to trace the course of axonal fibres. Several studies of this nature have reported a rostro-caudal gradient from human frontal cortex through caudate and putamen (Robinson et al. 2012; Verstynen et al. 2012; Jeon et al. 2014), and suggested a mirror caudorostral gradient from parietal cortex—see Fig. 7 (Draganski et al. 2008; Jarbo and Verstynen 2015). Corticostriatal tracts leading from S1, M1 and premotor cortex were found to overlap in caudal, motor striatum (Bohanna et al. 2011). Most recently, a specific examination of fibres from discrete sectors of posterior parietal, dorsolateral prefrontal and orbitofrontal cortex has identified a zone of 3-way convergence in the rostral body of the caudate and neighbouring putamen that is situated rostral to the motor striatum (Jarbo and Verstynen 2015), corroborating the presence of a folded topography.

In fact, dMRI methods not only capture the prevailing rostro-caudal topography of frontostriatal projections, but can also detect a significant asymmetry in this pattern, in that a higher density of fibres was identified projecting from human rostral cortex to caudal striatum than from caudal frontal cortex to rostral striatum (Verstynen et al. 2012). Equivalent patterns can be seen in comparing the striatal distributions of limbic, cognitive and motor compartments of the frontal lobe (Tziortzi et al. 2014), or the frontal sources connecting to successive rostro-caudal segments of the caudate (Kotz et al. 2013). These findings are consistent with a general formulation for the means by which behavioural control can propagate across the major BG domains, that relies on asymmetrical and non-reciprocal elements of circuitry (Haber 2003; Haber and Calzavara 2009). Equivalent experiments in monkeys, using anterograde tracers, show that limbic cortical areas (anterior cingulate and orbitofrontal) have focal projections to the rostral pole of the striatum, and more diffuse projections overlapping dorsolateral prefrontal (cognitive) input (Haber et al. 2006). This asymmetric pattern repeats itself with an invasion of striatal territory under the dominion of rostral motor areas (F7, SEF and FEF) by diffuse projections from cognitive areas (9 and 46) (Calzavara et al. 2007). Similar exchanges are achieved through striato-nigrostriatal and cortico-thalamocortical loops (Haber et al. 2000; McFarland and Haber 2002). There are, in effect, rostro-caudal cascades of BG loops and sub-loops (i.e. cortico-striatocortical^9^ and striato-nigrostriatal) mediating limbic/motivational influence over cognitive/planning stages that in turn feed through to premotor and motor cortices (Haber 2003; Haber and Calzavara 2009)—an observation in accord with broader ‘cognitive control’ theories of frontal organisation (Badre 2008; Badre and D’Esposito 2009).

Finally, it is worth noting the potential for another human imaging technique, fcMRI (functional connectivity MRI) to provide further insight into the nature of corticostriatal convergence. fcMRI charts correlations in slow oscillations of activity across the brain volume in the resting state. It thus infers connectivity, whilst specifying neither the direction nor directness of interconnection (Van Dijk et al. 2010). Several fcMRI studies have indicated that a single site in the striatum may couple (connect) with multiple, distributed regions of cortex (Di Martino et al. 2008; Barnes et al. 2010; Choi et al. 2012; Jung et al. 2014; Jarbo and Verstynen 2015). Alternatively, functional domains can be charted by assigning each striatal voxel to one of several alternative clusters, as determined by its maximal cortical coupling. This method has been used to segregate the striatal volume into five zones (Choi et al. 2012). Two of these are relatively discrete—one preferentially coupled to limbic cortex (in ventral striatum), the other to sensorimotor cortex (in posterior putamen)—whilst the remaining striatal territory forms three longitudinally extended zones, coupled to three distributed cortical networks (popularly known as the ‘default’, ‘frontoparietal control’, and ‘ventral attention’ networks, together forming a patchwork quilt over the frontal, parietal and temporal lobes) (Choi et al. 2012). It is important to note that the cortical networks reflect corticocortical coupling alone (Yeo et al. 2011), and that the winner-take-all strategy of assigning each striatal voxel to a single network visualises some relationships at the expense of others; for instance, the components of a sixth, ‘dorsal attention’ network (comprising posterior prefrontal (FEF, SEF), superior parietal and occipito-temporal cortex) are virtually eliminated from the striatal parcellation^10^ (Choi et al. 2012). Likely as not, the pattern of functional correlation will also be perturbed by active states, as opposed to the rest condition exploited by fcMRI. Thus, although no current parcellation of corticostriatal functionality aims to be definitive, it is clear that distributive associations can be identified, and future research will be capable of refining their functional characteristics and anatomical resolution.

### The ‘disclosed loop’ hypothesis

We are now in a position to resolve the ‘open’ vs. ‘closed’ characteristics of the cortico-BG loop. Strictly, the circuit as a whole is not closed, due to the initial corticofugal stage. As we have seen, there are various forms of corticostriatal convergence that reflect corticocortical associations. In particular, there is an asymmetric pattern of rostro-caudal convergence embedded within the core frontostriatal topography, upon which is superimposed longer range convergence from occipital, parietal and temporal cortices. By contrast, the corticopetal sector of the cortico-BG circuit *is* closed, in the sense that it is characterised by private, discrete output channels. The contrast between the corticofugal and corticopetal sectors is striking, as illustrated by one particular example: the locations of BG output neurons communicating with parietal area AIP, and premotor area PMv (F5), are notably separate (Clower et al. 2005) despite the fact that AIP and F5 are heavily interconnected (Borra et al. 2008; Gerbella et al. 2011) and share broadly convergent corticostriatal projections (Cavada and Goldman-Rakic 1991; Yeterian and Pandya 1993).

**Fig. 8:**
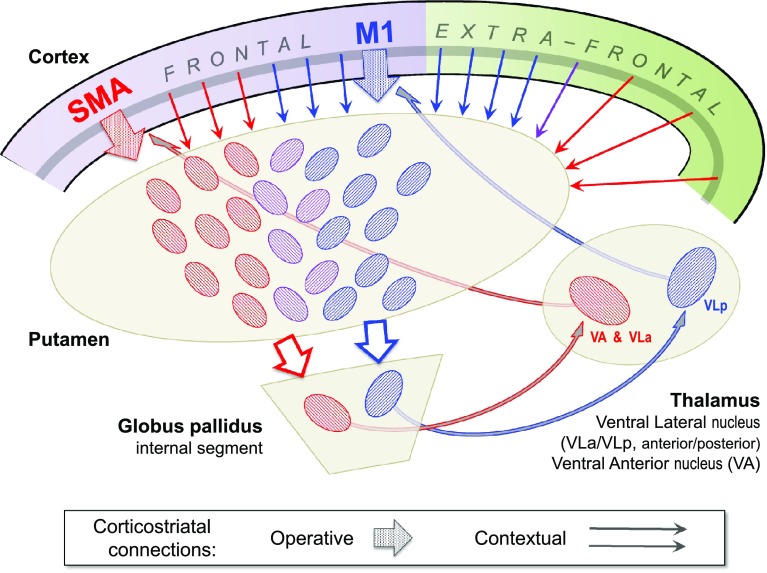
The disclosed loop model of the cortico-BG circuit. This is a schematic for the disclosed loop model of the *direct* pathway. The re-entrant sector of the BG pathway is mainly restricted to frontal cortex. It is composed of output channels (GPi–thalamus–cortex) that are topographically ordered and complete a closed circuit, here shown for example loops originating in M1 (*blue*) and SMA (*red*). Corticostriatal projections, by contrast, are highly divergent. Two classes are distinguished: operative (closed-loop) and contextual (open-loop). The operative afferents that issue from a specific site, e.g. a subunit of M1, innervate a set of matrisomes (shown as oval patches within the putamen) that converge upon the output channel in GPi that returns feedback to that same M1 subunit. The contextual afferents to a matrisome are those arising from cortex that does not receive feedback from the output channel to which that matrisome contributes. By definition, all corticostriatal afferents from extrafrontal (specifically, non-BG-recipient) cortex are contextual. Frontal afferents can be either operative or contextual. It is possible that a single afferent may perform both roles, as it passes through a large striatal territory and contacts multiple matrisomes. The divergence–reconvergence pattern shown by the cortico-striato-pallidal pathway can be pictured as a strategy to expose operative afferents to a broad range of contextual co-afferents in striatum, before the pathway converges back upon the appropriate output channel in GPi. The full details of these connections are not known. The schematic shows convergence of afferents from M1 and SMA in the lilac shaded patches, comprising an overlap zone of the M1 and SMA striatal input territories. Each of these patches represents a matrisome assumed to owe exclusive affinity to either the M1, or SMA output channel (as indicated by the slant of the patch). If so, SMA contributes some contextual input to the matrisomes feeding the M1 output channel, and vice versa. Other frontal motor areas known to contribute contextual inputs include CMAc, to M1 matrisomes, and PMd and PMv to SMA matrisomes (coded by small *blue*, and *red arrows,* respectively). Furthermore, it is plausible that M1 may mediate its own context (if some M1 afferents disrespect the somatotopic organisation of M1 output channels). S1 is the best documented source of extrafrontal contextual input to M1 matrisomes. The *blue*-*lilac*–*red* gradient of small *arrows* depicts a notional ‘folded’ topography of extrafrontal input to the striatum, as the identity of higher sensory/visuosensory areas specifically contributing to ‘*lilac*’ or ‘*red*’ matrisomes remains to be demonstrated. At a higher level of resolution, these definitions are more accurately applied to the input/output circuitry of individual striatal projection neurons (SPN). It is not known if the SPNs of a matrisome all feed the same output channel, or if a matrisome comprises SPNs with varied, single output channel targets; a third possibility is that each individual SPN might be capable of feeding multiple output channels

But that is not the end of the matter. Implicit in the term ‘loop’ is the notion of return to the starting point, and this in turn implies that among the convergent inputs funnelling into one BG output channel, there should be some obligatory contribution from the cortical target of that channel. This can also be framed as a more militant conjecture: that every single matrix output patch (matrisome) contributing to a given output channel should receive input from the cortical target of that channel. Although the conjecture acknowledges the open-loop architecture of BG circuitry, it echoes the closed loop in spirit, and relies on all the same anatomical evidence for support. For ease of reference the term ‘disclosed loop’ suggests itself: a refinement of the closed-loop formalism, with ‘disclosure’ indicating an open architecture at the corticostriatal stage. Figure 8 illustrates the principle and distinguishes ‘operative’ and ‘contextual’ corticostriatal output. Operative outputs establish the loop and arise from the cortical target of the BG output channel to which they contribute; contextual outputs arise from cortex that is not a target for the BG channel(s) to which they contribute. This anatomical distinction affirms the scheme of a bid for action selection and its contextual evaluation, raised in the Introduction—it is the operative output that launches the bid for selection.

The militant form of the disclosed loop thesis remains a conjecture, at present, because it is awkward to test experimentally. The nearest approach to date used anterograde and viral retrograde tracers placed at an equivalent cortical site (the arm representation in M1) in separate individuals, and compared the distributions of corticostriatal terminals with that of trisynaptic-retrogradely labelled striatal projection neurons (Kelly and Strick 2004). The result was a close match in the centre of gravity of the two distributions; failure to match exactly was not interpretable, due to the comparison being made across cases.

A corollary of the disclosed loop thesis that is more tractable anatomically offers better scope for refutation: that the entire volume of the striatum should receive input from some part or other of BG-recipient cortex. As noted above, known BG-recipient territory is currently frontal cortex plus post-Rolandic areas AIP and TE. Clearly, if some fraction of the striatum lacks input from this territory, its output cannot form a loop in sensu stricto. Does any such part exist? Frontal input to rostral and dorsal striatum (putamen, plus caudate head and body) is pervasive. The ‘folded’ topography here implies that input from non-BG-recipient parietal cortex cannot escape frontal convergence; for instance, patchy inputs from (parietal) S1 were always found to coincide with larger patches from (frontal) M1 (Flaherty and Graybiel 1995). The tail of the caudate, dominated by input from occipito-temporal cortex, is the most likely hiding place. The evidence here is sparser but the one detailed study largely supports the disclosed loop thesis: using retrograde tracers to study cortical transmission to the caudate tail, Saint-Cyr et al. (1990) comment that labelled cells in frontal cortex were “common to many or all” of the striatal injection sites. The equivocal phrasing reflected the fact that the three anomalous sites lacking evidence of frontal connections all used the same tracer (the dye ‘DY’ that had lesser sensitivity), and that in two of these cases, a second tracer (the dye ‘FB’) had a partially overlapping injection site and did produce frontal label (Saint-Cyr et al. 1990). A further consideration is that two frontal-anomalous striatal sites were connected to area TE, and the third to anterior parietal cortex (possible AIP), so these areas might alternatively satisfy the predicted input from BG-recipient cortex.

The frontal regions repeatedly noted to innervate the caudate tail were, jointly, the principle sulcus/anterior arcuate (FEF) region and anterior cingulate cortex (area 24) (Saint-Cyr et al. 1990). These observations tally with the origins of frontostriatal projections studied with anterograde tracer—specifically, the dorsal (large saccade) component of FEF (Stanton et al. 1988), and area 24c (Yeterian and Van Hoesen 1978). Certain areas of dorsolateral, medial and orbital prefrontal cortex have also been shown to extend projections to the furthest extremities of the tail (Yeterian and Pandya 1991; Eblen and Graybiel 1995; Ferry et al. 2000). Hence, the caudate tail retains the principle of pre- and post-Rolandic overlap demonstrated by the folded topography of more rostral sectors. The tail is dominated by signals from visual cortex; convergent inputs from dorsolateral, orbital and medial prefrontal cortex imply the additional influence of oculomotor planning and motivation. In short, this forms a potential example of an operative input, and its context. The next stage is to consider how context is evaluated, or in other words, how the striatum splits a bid for action selection into positive and negative salience signals for onward transmission through BG circuitry.

## Input–output architecture of the striatum

The disclosed loop thesis is that no part of the striatum lacks a component of operative input, conveying a bid for action selection. Its frontal lobe source expresses a continuum of decision making from emotionally based selection of behavioural priority to the physical minutiae of motor action. Each decision is governed by a host of factors (‘context’). The manner in which the circuitry of the BG loop may act to enforce competition and elect a victor remains uncertain. However, the striatum plainly assimilates many operational and contextual factors that might influence the outcome, and it is clear that the relative influence of these factors is plastic, their synaptic weights being subject to continual regulation by dopaminergic mechanisms reflecting the history of positive or negative reward outcomes from past actions. This, then, would appear to encapsulate the functional logic of the YVH or replication principle: corticostriatal convergence reflects the pattern of cortical network associations in order to capture an equivalent set of contingencies relevant to determining action. To consider the underlying neural mechanisms, it is necessary to introduce the functional architecture of the striatum.

### The evaluation of context

The output of the striatum arises solely from its dominant cell type, the GABAergic medium spiny projection neuron—or SPN—whose particular biophysics has been scrutinised in intracellular, in vivo recordings from rodents. A unique set of voltage dependent potassium channels act to hold the SPN membrane potential in one of two stable states, a non-spiking level of hyperpolarisation (‘Down’ state) or a more excitable (‘Up’) state induced and sustained by a barrage of excitatory glutamatergic input. Resting membrane potential in the Down state can approach −80 mV and is maintained by an inwardly rectifying potassium channel that resists small depolarisations, but inactivates in the face of more coherent inputs; this brings about the Up state, in which spiking is possible but not mandatory, and subject to neuromodulatory influences (Wilson and Kawaguchi 1996; Kreitzer 2009).

Corticostriatal axons provide this glutamatergic input mainly to the spines of SPNs, but distribute these contacts very sparsely. Each axon ramifies through a large territory of the striatum, dividing into a small number of long straight branches that form synaptic contacts en passant at relatively regular intervals (Parent and Parent 2006). Calculations based on the density and dendritic volumes of rat SPNs suggest that an axon would contact (via a single synapse) no more than 1 % of the SPNs within its striatal territory—and similarly, a pair of nearby SPNs would have no more than 1 % of afferent axons in common (Kincaid et al. 1998; Zheng and Wilson 2002). The cortical innervation of SPNs can be contrasted with that of one of the better studied class of striatal interneurons, the GABAergic, parvalbumin-positive fast-spiking interneuron (FSI). FSIs are known to receive direct cortical terminals and themselves to contact SPNs, forming a system for feedforward inhibition (Lapper et al. 1992; Bennett and Bolam 1994; Plenz and Kitai 1998; Silberberg and Bolam 2015). Axon reconstructions traced from sensorimotor cortex in the rat demonstrate multiple contacts (up to 6) from a single axon upon a single FSI (Ramanathan et al. 2002) suggesting that FSIs are rather more excitable than SPNs, in good accord with physiological observations (Mallet et al. 2005; Tepper et al. 2010; Paille et al. 2013). Also notable is the observation of direct convergence upon individual FSIs of afferent axons from the two cortical areas examined, M1 and S1 (Ramanathan et al. 2002). Remarkably, no study has yet attempted to replicate this anatomical observation for the output neurons themselves, SPNs; instead, evidence for convergence at the single cell level for SPNs obtains from cortical microstimulation, e.g. single neurons in putamen activated by dual electrodes, positioned at corresponding locations in the forelimb representations of M1 and SMA (Kaneda et al. 2002; Nambu et al. 2002a).

The biophysical specification of the SPN and its sparse innervation, coupled to the corticostriatal convergence described previously, has given rise to the accepted wisdom that individual SPNs will only activate when presented with sustained, synchronous inputs from a widely distributed and uniquely idiosyncratic subset of cortical sources. Hence, by virtue of detecting specific cortical states, SPNs have been considered to perform context recognition, computationally analogous to the threshold logic units of the ‘perceptron’ (a pioneering pattern classification network) (Houk and Wise 1995). The salience of the SPN’s signal to downstream structures would then depend upon the persistence, or stability of this particular cortical context. It is possible though that this picture should be replaced by one in which a single distal dendrite, rather than the entire dendritic tree, performs the necessary integration. Local release of glutamate appears to be capable of inducing a somatic Up state through regenerative activity confined to a single dendrite—and specifically its distal, rather than proximal elements—dependent upon NMDA receptors and voltage-regulated calcium channels (Plotkin et al. 2011). The principle of SPNs recognising the context of a particular cortical state may remain valid, but that context might be expressed by a far smaller ensemble of corticostriatal neurons. Furthermore, as the authors note, if (only) distal inputs to an SPN determine Up states, input to proximal dendrites may preferentially trigger spiking activity—as there is evidence that the induction of Up states and the initiation of spiking are synaptically independent (Stern et al. 1998; Plotkin et al. 2011). The ramifications of this model for SPN activation are explored more fully below (in the concluding ‘Functional Logic’ section).

### The regulation of trans-striatal pathways

As noted previously, there are two further sources of external input to the striatum, serving a more regulatory role. These are dopaminergic afferents from the SNc and ventral tegmental area (VTA) (Parent et al. 1983; Hedreen and DeLong 1991; Haber et al. 2000), and glutamatergic afferents from several thalamic nuclei, prominent among which are the ventral motor nuclei (McFarland and Haber 2000, 2001) and the intralaminar group (Smith et al. 2004; Sadikot and Rymar 2009). Helpfully, cortical and thalamic terminals can be distinguished anatomically by the presence of different glutamate transporters (vGlut1 and vGlut2, respectively), and whereas 95 % of cortical terminals so far identified are observed to contact spines (of presumed SPNs), the thalamic terminals are more evenly distributed between dendritic shafts as well as spines (Raju et al. 2008). In fact, the great majority of the nonspinous contacts onto dendrites are thought to originate specifically from the intralaminar nuclei of the thalamus, since all other thalamic sources that have been examined terminate selectively upon spines (Sadikot et al. 1992; Smith et al. 2009). These intralaminar afferents are also known to avoid the striosome compartments of the striatum, and to concentrate within the matrix (Sadikot et al. 1990; Sadikot et al. 1992).

A further important ultrastructural distinction between cortical and intralaminar thalamic input to the striatum is that dopaminergic terminals are found in close association with cortical terminals upon SPNs, but not with terminals of afferents from the intralaminar centromedian nucleus (Smith et al. 1994). Thus, dopaminergic regulation modulates the transmission of cortical signals, whereas the thalamostriatal system—or at least its intralaminar component—may operate through separate mechanisms. Rodent studies show that the intralaminar afferents also make specific contact with the cholinergic interneurons of the striatum (Lapper and Bolam 1992). This may serve an alerting function triggered by unexpected events, capable of interrupting striatal transmission (Smith et al. 2009; Ding et al. 2010).

### Differential regulation of the direct and indirect pathways

The basic formulation of the direct and indirect pathways marries their connectional status to a neurochemical signature: dSPNs express D1 dopamine receptors and their GABAergic transmission is characterised by peptide co-transmitters substance P and dynorphin; iSPNs express D2 dopamine receptors and use met-enkephalin as a co-transmitter (Gerfen et al. 1990; Graybiel 1990). D1 and D2 receptors couple with excitatory (G_s/olf_) and inhibitory (G_i/o_) G-proteins, respectively (Tritsch and Sabatini 2012), and consequently exert opposite modulatory effects over glutamatergic activation of SPNs, with both short and long term actions (Gerfen and Surmeier 2011; Surmeier et al. 2011). D1 receptors promote the transition to the ‘Up’ state of dSPNs and spiking activity; D2 receptors impede this transition and subdue spiking in iSPNs. This momentary regulation of SPN activity monitors the tonic level of dopamine afferent discharge, and is complemented by plastic changes of synaptic strength regulated by phasic dopamine signals (transitory peaks and troughs in the rate of dopaminergic discharge that reflect the presence and absence of reward (Schultz 2013). Phasic activation of D1 and D2 receptors promotes LTP and LTD (long term potentiation and depression) of glutamatergic synapses upon dSPNs and iSPNs, respectively; moreover, these actions are contingent upon recent spiking history, such that dopamine gates LTP or LTD of a synapse depending on recent conjunctions of pre-and post-synaptic depolarisation (Shen et al. 2008; Paille et al. 2013). As the underlying cellular mechanisms are not yet fully resolved in vitro, nor yet confirmed in vivo (Fino and Venance 2010; Pawlak et al. 2010), this account can be regarded as a viable working model of dopaminergic regulation, that also includes the complementary effects; induction of LTD in recently active dSPNs and LTP in iSPNs occasioned by a phasic decrement in dopamine signalling (Gerfen and Surmeier 2011; Surmeier et al. 2011).

It is the differential regulation of corticostriatal plasticity, coupled to the alternative output of SPNs to either the GPi/SNr or GPe that, in theory, enable the BG to fractionate a bid for action into positive and negative salience signals (Frank 2005; Hong and Hikosaka 2011; Schroll and Hamker 2013; Collins and Frank 2014; Baladron and Hamker 2015; Gurney et al. 2015). Take a scenario in whose context an operative signal activates a particular subset of dSPNs and iSPNs, and leads to reward: the outcome is to strengthen all the active inputs to dSPNs, and to weaken them to iSPNs. Alternatively, if the action leads to omission of reward, plasticity operates in the reverse direction. Hence, in any given context, a BG bid is processed by the activation of specific subsets of dSPNs and iSPNs and it is the balance of output transmitted along the direct or indirect pathways that determines whether an action is selected or restrained.

This picture of BG function is evidently built upon the foundations of the classic direct/indirect model. It extends it in assuming that the two classes of SPN share much the same input; or, in other words, that operative inputs divide equally amongst dSPNs and iSPNs, and that each class of SPN has access to the same range of contextual input, subject to plastic shaping by reward. Some studies in rodents (specifically, transgenic mice, in which dSPN and iSPN can now be readily identified—see below) indeed show that the subtypes of SPN are not readily distinguished by their inputs; apart from some variation in relative weight, the populations of cortical, thalamic and dopaminergic neurons contacting dSPN and iSPN are essentially similar (Wall et al. 2013). Individual dSPN and iSPN within the striatal matrix are found to receive equivalent proportions of convergent axospinous input from both cortical and thalamic sources (identified ultrastructurally, by VGlut1 and VGlut2) and, most significantly, single cortical and thalamic terminals are observed to contact spines of both SPN classes (Doig et al. 2010; Huerta-Ocampo et al. 2014). By contrast, there is some degree of preferential recruitment by two distinct types of corticostriatal projection neuron, referenced by their axonal characteristics—PT vs. IT (PT, branching into the pyramidal tract, or IT, remaining strictly intratelencephalic). Evidence from primate and rodent work has suggested that the IT component preferentially drives dSPNs, and the PT component iSPNs (Reiner et al. 2010). Functional differences of PT vs. IT sources might then allow some reconsideration of the role of the indirect pathway, and this is considered more fully below. First, however, there are potentially more fundamental concerns to address.

The classic direct/indirect model of BG function has resisted frequent challenges on the grounds that neither the neurochemical nor the connectional status of the striatal population of SPNs is quite as dichotomous as the model supposes (Bertran-Gonzalez et al. 2010). There is some evidence for blending in all respects—SPNs showing co-expression of D1 and D2 receptors, or the ‘wrong’ combination of peptides, or possessing axon collaterals to inappropriate targets. Confidence in the model has been boosted by recent behavioural studies in transgenic mice that exploit gene expression controlled by either the D1R or D2R promoter. Optogenetic applications in transgenic mice, for instance, enable selective stimulation of dSPN or iSPN populations, with opposing effects upon motor behaviour (Kravitz et al. 2010; Tecuapetla et al. 2014), operant reinforcement (Kravitz et al. 2012; Tai et al. 2012), or nigral or cortical activity (Freeze et al. 2013; Oldenburg and Sabatini 2015)—all largely confirming predicted outcomes. Hence, even if the proposed dichotomies are not absolute, they seem sufficiently preponderant to support the existence of two functionally distinct systems of striatal output. This is not to disregard the contrarian evidence, that repays further examination.

### Refinements in the anatomical identity of the direct and indirect pathways

Anatomical identification of separate source populations for the two pathways, initially achieved in the cat (Beckstead and Cruz 1986), is obtained by the use of dual retrograde tracers and depends on a low or zero count of double-labelling amongst interspersed populations of SPNs singly labelled by tracers placed separately in GPe and one of the output nuclei, either GPi (Gimenez-Amaya and Graybiel 1990; Flaherty and Graybiel 1993a), or SNr (Selemon and Goldman-Rakic 1990). However, retrograde tracers may have limited sensitivity to sparse collateral fields of axonal arborisation and it was later revealed by means of single axon reconstructions that many primate SPNs project to multiple BG nuclei in apparent violation of the direct/indirect model (Parent et al. 1995; Levesque and Parent 2005). The source neurons in these studies are not neurochemically classified as dSPNs or iSPNs—so can the distinction survive close inspection of their total axonal distribution? It was reported that 17 out of 21 reconstructed SPN axons originating from the matrix compartment of the striatum formed triple branches in SNr, GPi and GPe; the other four all had the GPe as a sole target (Levesque and Parent 2005). Significantly, the triply projecting axons produced the majority of their terminal boutons (approximately 60 %) in one of the two BG output nuclei, GPi or SNr—and so, with but a small fraction of their terminals (~20 %) within GPe, these axons ostensibly serve the direct pathway. By contrast, the four axons arborizing exclusively within GPe can be assigned to the indirect pathway. Thus, the two basic patterns of axonal collateralisation can be reconciled to the direct/indirect model (although the ratio of 17:4 is unexpectedly high, and requires further scrutiny). SPN axon reconstruction in the rat shows a similar pattern, in that all SPNs contact the GPe, but those that also contact SNr have a far smaller arborisation in GPe (Kawaguchi et al. 1990). More recent studies in transgenic mice have selectively manipulated axonal transmission from dSPNs to GPe, providing a functional rationale for these ‘bridging collaterals’ (Cazorla et al. 2014)—as reviewed below, in the ‘Functional Logic’ section.

The dual neurochemical identity of striatal SPNs originally established in rodents (Gerfen et al. 1990) has also been examined in primates. One initial report noted that the iSPN marker enkephalin was immunologically detected in 71 % of SPNs projecting to GPe, and 10 % of SPNs projecting to SNr (Flaherty and Graybiel 1993a). A second primate study used double in situ hybridisation (to detect mRNA transcripts) and found the expected co-expression of D1 receptor with substance P, and D2 receptor with enkephalin; the four possible crossover pairings (D1R/D2R, SP/enk, D1R/enk & D2R/SP) were also assessed, and in each case co-expression was estimated at about 5 % (Aubert et al. 2000). It would therefore be anticipated that the two populations of striatal SPNs would differentially contact GPi/SNr and GPe in accord with the direct/indirect model—but a subsequent primate study coupling retrograde tracing (from GPe or GPi) to immunolabelling of neurochemical markers gave several findings at seeming variance with this scheme (Nadjar et al. 2006). SPNs projecting to the GPe showed similar, high levels of D1 and D2 receptor expression—79 % and 87 %, respectively. Hence, the majority of these GPe-projecting neurons, 66 %, were of ambiguous status due to the implied co-expression of D1R and D2R.^11^ Similar results were obtained for SPNs projecting to GPi (73 % D1R and 74 % D2R)—and here, apart from the ambiguity created by implied co-expression, there was the added anomaly of SPNs expressing D2R alone projecting to a BG output nucleus (as 27 % of SPNs labelled from GPi had no visible expression of D1R) (Nadjar et al. 2006). Finally, combining retrograde labelling with expression of peptide markers in place of dopamine receptors gave a similar picture of unexpectedly high co-expression.

Nadjar et al. (2006) suggested that their results were at odds with the concept of a dual striatofugal system, and called for a reappraisal. That conclusion is not shared here. The foremost consideration is that if immunolabelling is more efficient than the mRNA methods employed by Aubert et al. (2000) at detecting marker co-expression, it may also be capable of detecting expression at levels that are not deterministic for the functional role of the cell. To draw an analogy with human sex hormones: in the absence of quantitative estimation, the mere detection of testosterone and oestrogen fails to distinguish gender. Thus, the fact that Aubert et al. (2000) reported negligible co-expression of the markers for dSPNs and iSPNs should not be discounted: serendipitous as it may have been, the sensitivity of the test looks to have been well matched to a level of marker expression that is indicative of two distinct classes of striatal SPNs. Nadjar et al. (2006) acknowledge that the actual level of co-expression may fall between the two estimates obtained by different techniques. They also note the possibility of retrograde labelling arising from ‘axons of passage’. This can be a substantial problem when the tracer used is one that is readily taken up by axons damaged by the injection syringe. The striatonigral bundle that courses through the pallidum en route to the substantia nigra is a very dense fibre tract (and indeed, contributes to the eponymous pallor of the structure) (Percheron et al. 1984). Hence striatal neurons labelled from either GPe or GPi might include SPNs projecting through the pallidum to SNc. This is a potential cause for SPNs labelled from GPi to show expression of D2R, rather than D1R as expected. Furthermore, at least in rodents, there is a subpopulation of SPNs concentrated within striosomes that shows co-expression of D1R and D2R (plus co-expression of the peptide markers substance P and enkephalin) known to project to SNc (Wang et al. 2006; Wang et al. 2007; Perreault et al. 2011). Finally, it is worth adding that a study of comparable design to that of Nadjar et al. (2006), but conducted in the rat, produced significantly smaller levels of inferred co-expression, and whose authors concluded that their results were well in line with the classic direct/indirect model (Deng et al. 2006).

### Differential drive of the direct and indirect pathways: PT vs. IT

Most of the known characteristics of the PT and IT subpopulations of corticostriatal neurons, including their differential input to the direct and indirect pathways, derive from work conducted in rodent area M1 (Table 1). In brief, IT neurons have a broader laminar distribution and communicate with bilateral cortex and bilateral striatum; PT neurons are largely confined to layer 5B, and their striatal collateral is a thin branch from a subcortical axon with numerous additional ipsilateral-only targets (including STN, thalamus and brainstem). The striatal arborisations of PT and IT axons look similar (sparse and expansive, as described above) (Parent and Parent 2006) but, under ultrastructural examination, PT axonal terminals are seen to be larger than IT terminals, about 50 % greater in diameter. The preferential distribution of contacts from IT to dSPN and PT to iSPN was first inferred from the size/frequency distribution of axospinous terminals upon the two types of SPN and supported—if with lesser bias—by direct identification of IT and PT terminals (IT terminals labelled from contralateral M1, and PT terminals labelled by tracer transported from the ipsilateral pyramidal tract) (Lei et al. 2004; Reiner et al. 2010; Deng et al. 2015). According to a computational modelling study, the physiological mode of synaptic transmission is another variable, IT to dSPN and PT to iSPN (the preferred contacts) being facilitatory, and the reverse contacts being depressive (Morita 2014). Hence, the relative drive imparted to each class of SPN may depend critically upon the time course of activity in the two corticostriatal populations (as depressive synapses have higher baseline probability of transmitter release). Transient optogenetic stimulation of IT inputs was shown to induce equivalent activation of dSPN and iSPN neurons, and PT stimulation actually gave a greater response of dSPN than iSPN—opposite to the anatomically anticipated bias (Kress et al. 2013). Selective electrical stimulation of IT inputs (via electrodes placed in contralateral motor cortex) also elicited equal spiking activity in dSPN and iSPN (Ballion et al. 2008).

**Table 1:** Characteristics of IT (intratelencephalic) and PT (pyramidal tract) corticostriatal neurons

Corticostriatal neurons	PT	IT
Classified by	Pyramidal tract axon	Intratelencephalic axon
Laminar location^a^	Layer 5, densest in 5B	Layers 2–6, densest in 5A and 3
Neural morphology^b,c,d^	Large pyramidal; profuse dendritic arborisation in layer 1	Medium sized pyramidal; sparser dendritic arborisation in layer 1
Laterality of axonal arborisation	Strictly unilateral	Frequently bilateral
Extrastriatal collateral^b,c,e,f^	Subthalamic nucleus, thalamus, brainstem, spinal cord	Cortex, claustrum
Striatal collateral	Thin collateral off main subcortical axonal trunk	Main subcortical axon
Striatal arborisation (l.m.)^f^	Scarce and widespread; longer terminals	Scarce and widespread; shorter terminals
Striatal terminals (e.m.)^a,g,h^	Large (50 % wider diameter)	Small
Preferential contact with striatal SPN^g,h^	dSPN 36 %	dSPN 54 %
	iSPN 64 %	iSPN 46 %
Short-term synaptic action upon dSPN^i^	Depressive	Facilitatory
Short-term synaptic action upon iSPN^i^	Facilitatory	Depressive

*l.m.* light microscope, *e.m.* electron microscope

^a^Reiner et al. (2003), ^b^ Wilson (1987), ^c^ Cowan and Wilson (1994), ^d^ Morishima and Kawaguchi (2006), ^e^ Kita and Kita (2012), ^f^ Parent and Parent (2006), ^g^ Reiner et al. (2010), ^h^ Deng et al. (2015), ^i^ Morita (2014)

If these observations afford some insight into the functionality of PT and IT outputs from M1, the picture outside M1 is more sketchy. PT corticostriatal neurons are widely distributed across frontal cortex (Feger et al. 1994) but in the remainder of the cortex they have only been positively identified within (rodent) somatosensory areas (Donoghue and Kitai 1981; Levesque et al. 1996; Reiner et al. 2010). As the cell bodies of PT corticostriatal neurons are largely confined to layer 5B (Cowan and Wilson 1994; Reiner et al. 2010), corticostriatal cells in all other layers (chiefly 5A and 3, as reported for primate) may be classed as IT by default (Arikuni and Kubota 1986; Saint-Cyr et al. 1990; Yeterian and Pandya 1994; Ferry et al. 2000; McFarland and Haber 2000). Comparisons of the laminar profile of retrogradely labelled corticostriatal neurons across sensorimotor cortex note a lesser concentration of cells in lower layer 5 of S1 relative to M1 (Jones et al. 1977; Wilson 1987), which implies that the frequency of PT corticostriatal neurons declines in S1. The same may be true for the corticostriatal outflow from the remainder of cortex in the parietal, temporal and occipital lobes—that it originates mainly from IT neurons, given that the laminar distribution of corticostriatal neurons looks similar in ipsi- and contralateral hemispheres (Saint-Cyr et al. 1990). However, as PT-type neurons (with outputs to thalamus, pons and tectum, if not the PT itself) are widespread throughout non-BG-recipient cortex, there is no obligatory reason to consider that corticostriatal PT neurons are absent.

What is known of the operational characteristics of IT and PT neurons that can help to interpret their potential differential drive to direct and indirect striatal outputs? Clearly, the absence (IT), or presence (PT) of an output to subcortical effectors (such as tectum, pons and spinal cord) encourages a distinction along a ‘planning–execution’ axis of motor control; the PT signal to the striatum might be considered an efference copy of the issued motor command. Functional properties of peri-movement activity recorded in primate M1 may illustrate such a distinction (Turner and DeLong 2000). The activity of identified IT corticostriatal neurons was triggered by very specific factors regarding the direction of a movement, or its time course. Some IT neurons responded solely to sensory stimulation. PT neurons (a nonspecific sample, lacking certified output to the striatum) were notably less selective. The activity of IT neurons was likened to that of the striatum itself, in the sense that it appeared to reflect specific contingencies regarding the production of a motor action. Now logically, ‘planning’ should be a precursor to execution, but here early onset (‘preparatory’) activity was common to both populations and IT activity did *not* systematically precede PT activity (Turner and DeLong 2000). The planning/execution distinction is better reflected by data obtained in paired intracellular recordings (Morishima and Kawaguchi 2006; Morishima et al. 2011), or by optogenetic stimulation (Kiritani et al. 2012) which shows that IT and PT neurons make plentiful recurrent connections amongst themselves, but that contacts from one population to the other are essentially one-way, from IT to PT. Thus, in terms of information, if not timing, PT neurons are downstream from IT neurons (Shepherd 2013).

### Toward a taxonomy of corticostriatal connections

We now have as many as five binary factors for categorising trans-striatal connections of cortical origin: they may arise from PT or IT neurons, either within or outside BG-recipient cortex; striatal targets may be dSPNs or iSPNs, located within striosome or matrix compartments; and the functionality may be operative or contextual. The systematics of striosomes is the easiest to excise from the implied permutations, since they impact only tangentially upon the substance of this review. As noted previously, striosomes process input from BG-recipient limbic cortex, and essentially feed the dopaminergic reward system rather than the return loop to cortex (Fujiyama et al. 2015). This includes striosomal SPNs with direct output to the SNc as well as to GPe, GPi and SNr (Levesque and Parent 2005). They might resemble dSPNs or iSPNs, but these striosome neurons possibly project to specific sub-loci within BG nuclei that participate in limbic circuitry, e.g. to a subclass of GPi neurons that project to the lateral habenula, a thalamic component known to form an inhibitory projection to SNc (Parent et al. 2001; Crittenden and Graybiel 2011; Hong and Hikosaka 2013).

The upshot is that the striosome and matrix compartments can be seen as acting in parallel but separate circuits, with the functional logic of direct and indirect cortical loops pertaining selectively to matrix function. Yet, having achieved some dimensional reduction by focusing upon the matrix compartment, a further factor deserves admission; termination of corticostriatal afferents upon the distal or proximal elements of the SPN dendritic field, as raised by Plotkin et al. (2011). In the total absence of specific anatomical data, this remains a hypothetical idea but it will serve as a useful tool in the concluding discussion to clarify how contextual/operative and PT/IT inputs may interact within the SPN dendritic field to determine the relative salience of cortical bids for action selection via BG circuitry.

## Functional logic of the disclosed loop

We have seen how the operations of BG circuitry can be considered to enact a competition between rival bids for action selection. The term ‘action’ befits circuits looping through motor cortex, but can also stand more abstractly for any subunit of decision-taking across the broader emotional and cognitive competence of the frontal lobes. The structure of the BG loop justifies the oxymoron that it is both open and closed, in that the corticofugal connections leading to the BG input nucleus, the striatum, are highly divergent whilst the corticopetal pathways, also known as BG output channels, are far more focal in their topographic organisation. The anatomical formalism of the disclosed loop is that every sector of the striatum—indeed every SPN—should receive some input from the specific zone of cortex that is targeted by the BG output channel(s) to which that SPN contributes, thus establishing a loop in sensu stricto. The functional logic of the disclosed loop is that this input, here termed an ‘operative input’, establishes the bid for action selection; the salience of the bid in its onward transmission through BG circuitry is determined by the broader range of contextual inputs to the striatum, deriving from frontal and non-frontal cortex irrespective of the reception of BG feedback. The distributed origin of contextual inputs that converge upon a given site in the striatum mirrors the transcortical network harnessed by the source of the operative input to that site, and thereby captures a similar range of contingencies relevant to determining action. Finally, the positive and negative aspects of bid salience are decoupled in the striatal origins of the direct and indirect pathways. Operative input is fed to both components by individual corticostriatal terminals contacting both direct and indirect SPNs; similarly, contextual input is available to each subsystem alike, but is shaped by a differential history of reward outcomes from past actions, effected by dopaminergic regulation of corticostriatal plasticity.

This much emerges from a focus upon the input/output architecture of the striatum, as reviewed above. The distinction between operative and contextual additionally draws upon a more panoramic view of BG circuitry, and deserves further consideration in relation to previous conceptions of BG circuit organisation. Several other fundamental issues of BG function depend rather more critically upon the precise organisation of extrastriatal microcircuitry. These include: (1) the level of action specificity to be expected of the ‘microchannel’ invoked by network computational models, and how it may be governed by the gross funnelling between successive BG stations; (2) the integration of direct and indirect pathways within the BG output nuclei; (3) the neural nature of competition between rival bids, and (4) the functions associated with multiple triadic sub-loops and reciprocal connections between BG nuclei. Equally, stepping in the opposite direction to circuit macro-architecture, consideration of the adaptive mechanisms of corticostriatal plasticity depends upon an analysis of intracellular signalling systems. Perforce, the following discussion must skirt around these topics. Instead, it will address two main questions. First, the potential combinatorial effects upon SPNs offered by the diversity of corticostriatal sources—reflecting both tangential (operative/contextual) and radial (PT/IT) cortical organisation; secondly, how the current status of the construct of direct and indirect pathways hinges upon the characteristics of dSPNs and iSPNS, and what computational advantage is offered by such a schism. To begin, it is useful to pursue the implications of a recent reappraisal of SPN cellular biophysics.

### Functional architecture of the striatal projection neuron

As noted above, pioneering in vitro manipulation of rodent SPN has now shown that the distal elements of dendrites are capable of regenerative activity, and the independent induction of an Up state recorded in the SPN soma^12^ (Plotkin et al. 2011). In consequence, the number of cortical (and/or thalamic) afferents responsible for generating an Up state may be far smaller than previously thought. Indeed, the authors of this study estimate that synchronous activity on the part of 12–15 corticostriatal pyramidal neurons, firing at typical in vivo rates and converging upon a 20 μm stretch of a single distal dendrite could be sufficient. The dendritic tree of the SPN might thus constitute multiple functional subunits, each capable of detecting a different state of cortical activity. The authors go further to suggest that separate subsets of afferents may be responsible for inducing Up states, and for triggering spikes; the latter set would evidently include contacts upon proximal SPN dendrites in addition to (or, possibly, to the exclusion of) distal contacts. For ease of reference, let us refer to this as the ‘PDS’ model of SPN biophysics. It immediately confers a richer functional insight into the disclosed loop thesis.

Adopting the PDS model, it would be natural to suppose that contextual inputs to distal SPN dendrites act to generate Up states, and that SPN spiking reflects the timing of operative input. Contextual input does not, by itself, drive the SPN to fire, but acts in a gating role to permit operative inputs to do so. Hence, the efficiency of the operative drive is conditioned by the frequency with which it coincides with a contextually primed Up state. The PDS model of the SPN thereby conjures a physiological dimension to the anatomical distinction of ‘operative’ and ‘contextual’ input.^13^

### More dualities: PT vs. IT and dSPN vs. iSPN

IT and PT afferents from frontal cortex are held to convey information relating to planning and execution of actions, respectively, on the grounds that PT (but not IT) afferents are collaterals of axons descending to brain stem effector nuclei; also, in that the population of PT corticostriatal neurons is downstream from IT corticostriatal neurons in the processing of cortical signals (Reiner et al. 2010; Shepherd 2013). ‘Planning’ is certainly an appropriate metaphor to characterise operative IT afferents. It can also be consistent with the provision of context; this would be the most likely interpretation of the transcallosal contingent of IT afferents, for instance, where right- and left-sided actions are potentially in conflict, or require coordination. Context is also the role ascribed to sensory/associative IT input from extrafrontal, non-BG-recipient cortex. PT inputs are equally likely to convey context, for example, in the control of action sequences, where the state of the current action is an important factor in the selection of the upcoming action. The context associated with the asymmetric ‘cascade’ architecture of corticostriatal gradients described above, whereby prefrontal limbic and executive influences are progressively brought to bear upon motor control (Haber and Calzavara 2009) could be implemented by both IT and PT inputs.

Performance of an operative role by PT inputs presents a temporal paradox: how can the instruction to execute an action participate in its prior selection? One solution is to propose that an initial phase of PT activity is subthreshold for motor action (e.g. akin to ‘buildup’ activity in collicular neurons; Munoz and Wurtz 1995). Alternatively, the role of operative PT input may be to sustain peri-movement SPN activity to lock out rival actions. Indeed, SPNs are commonly observed to remain active across pre-, peri- and post-action periods (Lau and Glimcher 2007). A third and final consideration is that PT operative input could continue to function post-action to govern plasticity. Reinforcement learning theory holds that the sign of modification of corticostriatal synaptic efficacy (LTP vs. LTD) depends upon several interacting signals; these include an eligibility signal denoting recent synaptic activity, an outcome signal denoting gain or loss of reward, and an action signal denoting whether or not the action was selected and performed (Redgrave and Gurney 2006; Izhikevich 2007; Fee 2014). Operative PT afferents, by acting as an efference copy, could provide the action signal.

None of this theorising yet provides a rationale for either operative/contextual or PT/IT input to differ between dSPNs and iSPNs. Quite the opposite, in fact: the functional principles governing the PT/IT sources of contextual and operative inputs, and their dendritic contacts should apply to both classes of SPN alike. It is therefore appropriate to emphasise that all four variants of corticostriatal transmission (IT/dSPN, IT/iSPN, PT/dSPN and PT/iSPN) have been experimentally demonstrated (Ballion et al. 2008; Kress et al. 2013). On the other hand, this does not obligate strict anatomical uniformity, and preferential contacts of IT/dSPN and PT/iSPN are established in both rodents and primates (Reiner et al. 2010; Deng et al. 2015). Various functional rationales have been proposed to account for the asymmetry. One that gels with the disclosed loop thesis is that PT input—specifically operative PT input—has a particular affinity with iSPNs for the purpose of action termination (Reiner et al. 2010). The FEF, for instance, issues saccadic commands in retinotopic coordinates and an overly prolonged discharge might cause a double eye movement with the same vector. If the indirect pathway indeed acts to terminate motor commands, the time course of iSPN spiking should outlast the opponent dSPN spiking, being sustained by operative PT input alone as operative IT inputs cease. Again, this is what the physiological evidence indicates—that selective stimulation of PT (or IT) afferents is sufficient to drive spiking activity in iSPNs (or dSPNs) (Ballion et al. 2008; Kress et al. 2013).

In this scheme for action termination, the PDS model of an iSPN might predict operative PT contacts onto distal dendrites as well as proximal dendrites, to generate the necessary Up states. A role in the regulation of plasticity might mandate a similar dendritic distribution to enable independent drive by operative PT afferents carrying an ‘action signal’—but in that case upon both classes of SPN. Considerations of this nature challenge the notion of a strict allocation of operative and contextual inputs to proximal and distal dendrites respectfully. Is that, then, an over-specific interpretation of the PDS model of the SPN? Can we envisage a complementary rationale for contextual inputs to contact proximal dendrites and trigger spiking activity? The following section entertains such a scenario.

### Synaptic role-reversal of operative and contextual inputs?

A recent model of corticostriatal plasticity incorporating the ‘action signal’ noted above also stipulates distinct roles for contextual and efference copy inputs to an SPN (Fee 2014). Drawing on the avian homologue of BG circuitry, it proposes that the BG loop is not directly engaged by exploratory actions and behaviour, but gains the capacity to bias cortical decisions through reinforcement learning resulting from these actions. In this formulation, synapses made by contextual afferents, alone, drive SPN spiking and are capable of undergoing plastic changes. The role of efference copy afferents is to gate plasticity, in their role as an action signal, notionally through the induction of a dendritic Up state (Fee 2012, 2014). This scheme readily translates into the disclosed loop/PDS model discussed above, but with reversed specificity: contextual afferents should contact proximal SPN dendrites to trigger spikes, whilst efference copy equates to operative input, now directed to distal dendrites. For shorthand (and from the current perspective) we can refer to it as the ‘role-reversal’ model.

The deposition of a focal, driving output from motor cortex to striatum, to establish a bid for action selection, is a mainstay of network models of BG function (Redgrave et al. 1999; Gurney et al. 2001a; Schroll and Hamker 2013). Microstimulation experiments indeed confirm that motor output has this spike-triggering capacity (Nambu et al. 2002a; Ballion et al. 2008). Yet the role-reversal model places the onus for action selection entirely with the broader origins of contextual input—sufficient for some circumstances, as illustrated by a schematic of a cue-driven oculomotor choice task (Fee 2012, 2014). So what aspects of BG functionality are lost with the omission of a driving, operative input in the role-reversal model?

One obvious loss is the ability to encode context-free fluctuations in the reward value of an action. A free-choice task, with asymmetric reward for two alternative actions (a right or left handle movement), provides evidence of the requisite operative plasticity. The monkey’s behaviour is shaped through trial and error learning alone, as no cue is provided as to which action will earn greater reward (Samejima et al. 2005). The design of this task sets up a contest between rival bids for right or left action selection. These trial actions are learnt and reinforced in the context of the lab environment, and the primate chair—but this context is identical for both actions. Hence, context alone cannot select the optimal action for reward. Individual SPNs are said to encode ‘action values’, as their activity waxes and wanes as the high reward action is switched across blocks of trials (Samejima et al. 2005; Ito and Doya 2011). The inferred neural mechanism requires bidirectional plasticity of a driving, operative motor planning synapse.

A second feature of the role-reversal model is its reliance upon the closed-loop characteristics of re-entrant BG circuitry to target the appropriate cortical command centres. In this respect, it shares a common platform with network models that assume microchannels with action specificity. Plainly, ‘actions’ are characterised by a continuum of muscular forces and kinematics, and some anatomical degradation of action specificity is to be expected in the convergent funnelling from cortex through to the BG output nuclei (Brown et al. 2004). Contextual drive to SPNs could determine the net firing rate of BG output nuclei, and emulate the tonic thalamic disinhibition achieved within network, rate-coding models (Schroll and Hamker 2013). Operative drive to SPNs, by contrast, would allow subtler dynamical variations, issuing from the focal origin of the loop, to influence the effective connectivity of the re-entrant BG circuit. Coupled oscillations have been found between cortex and striatum (Courtemanche et al. 2003; von Nicolai et al. 2014), and at subsequent BG stations (Leventhal et al. 2012), that are modulated during behavioural tasks. Such physiological mechanisms could quite plausibly sharpen the effective action specificity of the re-entrant BG circuit, and this would depend upon an operative driving action upon striatal SPNs, as envisaged by the disclosed loop/PDS model.

### The status of the direct/indirect pathway model

The distinction between twin classes of SPN is critical to the classic model of dual BG loops, and has been amply confirmed—in mice—by the advent of bacterial artificial chromosome (BAC) transgenics (Valjent et al. 2009). This technology has been exploited to place transgene expression under the control of the D1 or D2 receptor promoter, either to identify dSPN or iSPN by expression of fluorescent labels or to permit selective optogenetic stimulation. Initial optogenetic manipulation demonstrated the functional opponency predicted by the classic model, in that bilateral stimulation of dSPN enhanced free locomotion (or contraversive turning, if delivered unilaterally) whilst stimulation of iSPN promoted freezing (or ipsiversive turning) (Kravitz et al. 2010). Research in this vein continues apace supplemented by techniques for monitoring identified dSPN and iSPN activity during active behaviour (Cui et al. 2013; Isomura et al. 2013). Not all results are so supportive of the classic model, nor predicted by it. Perhaps inevitably, there is some friction in reconciling all manifestations of functional complexity in BG circuitry to the elemental principle that the direct pathway promotes and the indirect pathway activity restrains action. Lingering unease with the classic model builds upon evidence for cross-talk between the direct and indirect pathways (either by bridging collaterals to the GPe, or by cross-neuromodulatory regulation mediated by intrinsic striatal circuitry) to propose that dSPN and iSPN each share a joint responsibility to initiate, and restrain movement (Calabresi et al. 2014). Ultimately, the ambition of the research effort is neither to bury nor to praise the classic model, but to refine it. The disclosed loop thesis, developed here as an extension of the classic model, should inform the debate and provide a sharper tool for the dissection of ambivalent evidence.

**Fig. 9:**
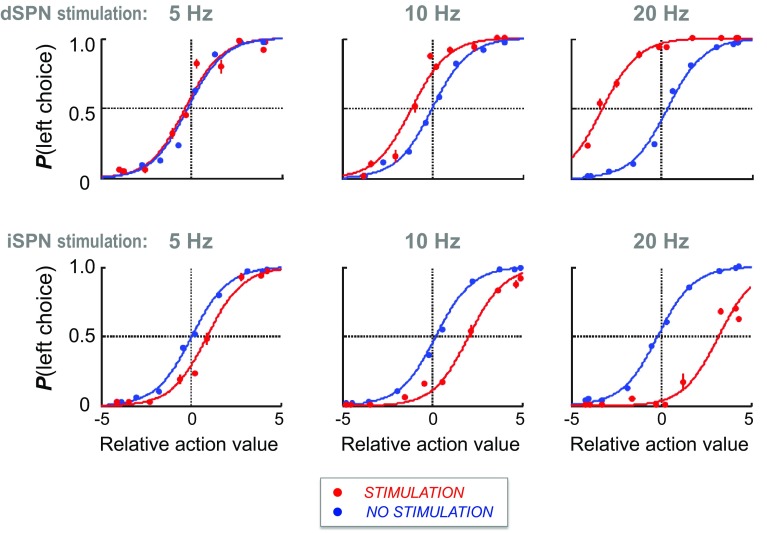
Optogenetic activation of SPN mimics a change in action value in a two-alternative, forced-choice task. Trials are initiated by a ‘Go’ cue, after which mice select a right or left port for a nose press; reward is asymmetric, and the rewarded side is switched across blocks of trials, with no cue for guidance. Individual choice behaviour can be modelled from the running history of recent choices and rewards, computed as a relative action value for the two available choices. Unilateral optical stimulation of SPN is applied at the time of the Go cue, in a small fraction of trials; it influences action selection, as shown by the plots of fractional choice for the left port against relative action value immediately prior to each choice. The curves are a logistic fit to the data obtained in control (*blue*) and stimulation (*red*) trials, the latter showing variable horizontal shift dependent on three different intensities of optical stimulation. As each hemisphere governs contralaterally directed behaviour, stimulation of dSPN in right dorsomedial striatum is modelled as an additive effect upon the action value of a left choice (*upper panels*; leftward shift of *red curve*); stimulation of iSPN in right dorsomedial striatum as a subtractive effect upon the action value of a left choice (*lower panels*; rightward shift of *red curve*). Redrawn, by permission of Macmillan Publishers Ltd: [Nature Neuroscience] (Tai et al. 2012)

The emerging picture is that dSPN and iSPN capture the positive and negative components of the salience of a bid for action selection, as expressed by their relative activity during a ‘planning stage’ of behaviour. Salience is determined by the intensity of the operative signal (submitted equally to dSPN and iSPN) as gated by the prevailing context. Contextual synapses are surmised to regulate the frequency of SPN Up states, subject to plasticity conditioned by the history of reward. The synapses mediating operative drive are also inferred to be plastic, as observed by Samejima et al. (2005): this study (outlined above) uses the behaviourist term ‘action value’ in place of the more mechanistic notion of ‘salience’, but the underlying rationale is much the same; notably, it reports a similar incidence of SPNs with positive and negative action values. The respective identification of these classes as dSPN and iSPN has now been buttressed by optogenetic manipulation within a similar operant paradigm (Tai et al. 2012). This experiment offered mice asymmetric reward for right or left nose-pokes, the direction of reward reversing between blocks of trials. Under this regime mice followed a ‘win-stay, lose-shift’ strategy that was perturbed by optical stimulation of dorsomedial striatum, applied in a small fraction of trials (6 %) to coincide with the ‘Go’ cue. This intervention did not compel a right or left choice but exerted a probabilistic effect by causing a change in relative action value, according to a computational model of behaviour (see Fig. 9). Unilateral stimulation of identified dSPN or iSPN had an additive or subtractive influence, respectively, upon the action value of a contralateral poke (Tai et al. 2012; Lee et al. 2015).

What part do operative and contextual inputs play in this scenario? Trials were initiated by illuminating a ‘Go’ cue, after the nose was positioned at the centre port, but there was no further contextual cue to guide choice behaviour, or to indicate a switch of reward across blocks of trials. The sensorimotor contextual input to SPNs at trial initiation should come to prime the operant response, but to do so equally for right or left choice. Hence, as with the study of Samejima et al. (2005), it is the state of the operative input to SPNs, undergoing plastic change through dopaminergic reward mechanisms, that is inferred to determine a choice between two rival bids for action selection.

Now, if SPN stimulation at the time of the ‘Go’ cue mimics a different state of operative transmission, shifting action value (Tai et al. 2012), can SPN stimulation at a later, post-action phase, mimic the effect of dopaminergic reinforcement, and influence task-learning? To test this idea, bilateral optogenetic (laser) stimulation of dorsomedial striatum was triggered by an operant touch sensor: given a choice of triggers, auto-stimulation of dSPN produced a bias toward the laser-paired trigger in one group of mice, whereas auto-stimulation of iSPN in a separate group produced a bias away from the laser-paired trigger (Kravitz et al. 2012). Once again the differential effect (here, reinforcement vs. punishment) is in accord with the classic model. No overt reward was given for trigger press, and a combination of D1R and D2R antagonists had no effect upon the outcome. In theory, therefore, the additional spiking caused by optogenetic stimulation may have been sufficient to emulate the effect of the dopaminergic reward signal upon SPN activity (Collins and Frank 2014)—either the phasic peak, or dip, in dopamine release known to enhance dSPN and iSPN activity, respectively (Gerfen and Surmeier 2011; Surmeier et al. 2011). If so, the emulation of a positive dopaminergic signal by dSPN stimulation may have been more ‘physiological’, as the negative trigger bias was only a transient learnt effect, whilst the positive trigger bias was more persistent (Kravitz et al. 2012).

### Why is there a dual (bipolar) encoding of action value?

The capabilities of the dopaminergic regulatory system provide a computational rationale for the division between dSPN and iSPN of positive and negative controls upon action; intuitively, at the very least, the dual system allows for a more flexible regulation of behaviour (Collins and Frank 2014). To expound the argument, the effect of SPN stimulation prior to action selection (Tai et al. 2012) can also be interpreted as an emulation of dopaminergic signalling—in this case the *tonic* dopaminergic signal^14^ (Collins and Frank 2014). The latter is associated with ‘incentive’ theories of dopamine, concerning the role of dopaminergic tone in the motivation and vigour of behaviour (Berridge 2012); note that stimulation of SPN prior to action influenced the vigour of choice behaviour, as well as its direction (Tai et al. 2012). Therefore, dopamine dynamically regulates the balance of action values: whatever the plastic state of the operative synapse, or the momentary context, tonic dopamine release effectively enhances the estimation of gains and attenuates the estimation of losses with regard to action selection (Collins and Frank 2014). This is ecologically significant as the cost/benefit analysis for any given action is not a constant for any given contextual environment; external risks may matter more, or matter less, dependent on internal state.

The neural dimension to this discussion is wholly conjectural. If the direct and indirect pathways were to be amalgamated, a hypothetical alternative striatal architecture would feed all operative and contextual input to a single class of SPN. Since these glutamatergic inputs are excitatory, negative context must then be signalled via interneurons—perhaps through presynaptic dendritic terminals such that single interneurons act as multiplexors, performing local sign reversal for multiple axonal contacts, as exists for thalamus (Crandall and Cox 2012). Clearly, any such a reduced system must lack the computational capacity for dopaminergic regulation provided by the existence of separate classes of SPN, in which cellular and dendritic mechanisms of signal integration surpass the limitations of single spines and synapses.

### Anomalies in direct and indirect pathway signalling: dSPN and iSPN co-activation

An observation could be considered anomalous if it conflicts with a model of BG function—which is not unexpected, given that even the most sophisticated models are subtotal assimilations of known BG circuitry, which itself is incomplete. Opinions can differ, however, on what does or does not conflict, even with the classic model. For instance, recent methods for observing (rather than stimulating) activity in identified dSPN and iSPN have demonstrated a simultaneous co-activation of the two classes whilst performing an operant key-press, that typically preceded movement onset by up to 500 ms and was greater for contraversive movement (Cui et al. 2013). This finding met with an ambivalent reception—regarded as a challenge to the classic model, but perhaps reconciled to it if the co-activation of the indirect pathway were for the purpose of inhibiting rival actions (Cui et al. 2013; Calabresi et al. 2014; Nelson and Kreitzer 2014; Wang et al. 2015). Such views appear to overlook the role of the indirect pathway in registering the negative context of a planned action, as reviewed above. Co-activation of dSPN and iSPN pertaining to the *same* action can be seen as an expected consequence of the fact that single corticostriatal terminals contact both classes of SPN (Doig et al. 2010), whose relative activity is set by the balance of positive and negative context. Hence, anti-kinetic (iSPN) pre-movement activity (Cui et al. 2013) might represent concurrent opposition to the actual performed action as well as notional rival actions. To pursue the question further, it is useful to reconsider BG circuitry beyond the striatum.

As outlined by the Introduction, there are at least two modes by which the indirect pathway may subdue action selection. One is a blanket suppression of all action, utilising diffuse excitation of the BG output nuclei by the STN, assuming that the disinhibitory chain from iSPN to GPe to STN augments prior activation of STN by cortex. The second is a more focal suppression of specific actions mediated by the short limb of the indirect pathway (iSPN–GPe–GPi/SNr). In the experiment of Cui et al. (2013), mice performed a two-lever free-choice task and were rewarded every ten presses, left or right regardless. The context of the operant apparatus would elicit motor planning for lever pressing, dominating the operative input to dSPN and iSPN. But right and left press would be rival action plans, each expressing negative context for the other (e.g. via crossed IT corticostriatal afferents contacting iSPNs) with the consequent potential for specific cancellation via the shorter, focal limb of the indirect pathway. This would be sufficient to account for the observed co-activation of dSPN and iSPN in the same hemisphere, simultaneously promoting and restraining one and the same action. Latent planning for alternative behaviours (e.g. exploratory whisking) would fall victim to blanket suppression mediated by the STN limb of the indirect pathway; there should be little or no sustained operative drive to SPN for these alternative behaviours.

In summary, from the perspective developed here, co-activation of dSPN and iSPN is not of itself an anomaly—although there are certainly open questions about the relative activities leading to action selection vs. cancellation, and the dynamics of signal integration within the output nuclei. Specific action cancellation requires action planning in the first place, but this is simply to assert that iSPN (and dSPN) spiking reflects the activities that are performed in a given operant setting, as opposed to other elements of behavioural repertoire that are neither planned, rewarded nor observed.

### The ‘anomaly’ of direct pathway bridging collaterals

Bridging collaterals—the axonal branches of dSPN terminating within GPe—have been considered inconsistent with the classic model because they link the two striatofugal pathways that canonically are separate and independent (Levesque and Parent 2005; Calabresi et al. 2014). One means of reconciling bridging collaterals to the classic model is to suppose that they act to suppress alternative actions. There is indeed some evidence in favour of this proposition, obtained in mice from the effect of bilateral optogenetic stimulation of dSPNs upon locomotion, coupled to electrode recordings in GPe, or SNr (Freeze et al. 2013). Optical stimulation of dSPN gave rise to both inhibition and excitation of SNr activity. The inhibition was registered at short latency (median 20 ms) consistent with direct input from the striatum, whereas the latter effect, excitation, occurred at longer latency (median 60 ms) matching the latency of SNr excitation achieved by iSPN stimulation (Freeze et al. 2013). Thus, the presence and timing of SNr excitation following dSPN stimulation is consistent with an indirect relay of dSPN signals to SNr via GPe. Importantly, the dSPN stimulation produced excitation and inhibition in different SNr neurons (and not at different times in the same neurons) (Freeze et al. 2013)—as would be expected if the dSPN main axons and their collaterals to GPe influenced the activity of separate populations of BG output neurons.

The density of the dSPN projection to GPe has been found to be remarkably plastic in adult mice, and to be governed by the excitability of iSPNs^15^ (Cazorla et al. 2014). This study employed the same optical stimulation of dSPN (bilaterally, in dorsomedial striatum) and replicated its positive effect upon open field locomotion in control animals (Kravitz et al. 2010; Freeze et al. 2013). Conversely, however, dSPN stimulation was found to *inhibit* locomotion in mice treated to develop enhanced collateral transmission from dSPN to GPe; furthermore, this behavioural effect was accompanied by greater inhibition of GPe neurons, at a latency mirroring stimulation of iSPNs (Cazorla et al. 2014). These findings thus demonstrate, in principle, that dSPN bridging collaterals can act to mimic the indirect pathway and restrain movement. It is also possible that the direct and indirect relays of dSPN signals to SNr may target separate populations of SNr output neurons. Such anatomical specificity could account for two separate populations of nigrotectal neurons, studied in cat (Jiang et al. 2003). Crossed and uncrossed nigrotectal neurons show remarkably different physiological properties. These are listed in Table 2, but in summary all the atypical properties of crossed nigrotectal neurons are consistent with the proposal that they receive dSPN input via bridging collaterals to the GPe, to inhibit saccadic commands by the contralateral SC—exactly opposite to the facilitative role played by uncrossed nigrotectal neurons (Wurtz and Hikosaka 1986).

**Table 2:** Characteristics of crossed and uncrossed nigrotectal neurons in cat SNr (Jiang et al. 2003)

Nigrotectal neurons:	Uncrossed	Crossed
Antidromically activated	Only from ipsilateral SC	Only from contralateral SC
Spontaneous activity	Relatively high: 36.8 ± 18 Hz	Relatively low: 12.5 ± 10 Hz
RF size	Relatively small	Relatively large
Response to visual stimulation	Phasic inhibition	Phasic excitation
Receptive field (RF) location	Congruent with RF in target (ipsilateral) SC	Incongruent with RF in target (contralateral) SC
Topographic distribution of nigrotectal terminals	Relatively focal	Relatively broad

These properties suggest contrasting but complementary roles for the crossed and uncrossed nigrotectal projections; the former to achieve blanket suppression of saccades to any location in the hemifield ipsilateral to the recorded SNr, and the latter to facilitate a saccade to a specific location in the contralateral hemifield. If crossed nigrotectal neurons are indeed modulated by dSPNs via the GPe, they can be considered to implement a ‘crossed direct’ pathway

If this interpretation of bridging collaterals retains them within the fabric of the classic model, it risks violating another principle: that of the closed re-entrant loop, pertaining to the pathway from SPNs back to cortex. If dSPN collaterals leak information between BG loops in this way, does that affect the definition of an operative input to a SPN that is contingent on the eventual cortical target of BG feedback? To revisit these issues, it is necessary to bring the discussion back to its own starting point.

### Leaks in the disclosed loop?

A closed re-entrant organisation is indicated by the sum of transneuronal retrograde studies (reviewed above) which show that the BG output module, GPi/SNr, communicates in a point-to-point fashion with frontal cortex. The functional specificity of a BG output channel depends on the means by which signals funnel into it through trans-striatal connections from the cortex. As shown in Fig. 8, this may follow a divergence–reconvergence strategy—the output from a focal zone of cortex diverges to a set of discrete patches within the matrix compartment of the striatum (matrisomes) that then re-converge upon a single output channel. This was anatomically demonstrated by dual-tracer studies in primate, relating to the foot area of sensorimotor cortex (S1 or M1 alike); striatopallidal convergence was similar, whether directed toward GPi or GPe, thus replicating the divergence–reconvergence organisation across both the direct and indirect pathways (each served by matrisomes containing a mixed population of dSPN and iSPN) (Flaherty and Graybiel 1993a, 1994). In consequence, an operative input to a matrisome (or to a single SPN) can be defined as one that forms the closed loop, whilst the purpose of the open-loop afferent architecture is to recruit additional, contextual inputs. Broad corticostriatal divergence acts to extend the combinatorial context to which an operative signal is exposed (Flaherty and Graybiel 1994).

To refine the classic model, dSPN bridging collaterals and iSPN afferents that share the same operative drive might target either the same or different populations of GPe neurons. In the first case, the direct pathway would operate with the handbrake permanently on (a somewhat counter-intuitive possibility). The second case is the one envisaged in the previous section, where one sub-element of the direct pathway applies the brake to other elements; this is more in keeping with the classic model, as it constitutes a form of competition between rival bids for action selection. Either way ‘operative’ is still understood to refer to the subset of corticostriatal contacts that establish a closed loop, though a formal definition should distinguish a class of SPN and the action of feedback, viz: ‘operative input to a dSPN is that input received from the specific zone of cortex that receives positive feedback from the BG output channel(s) to which that dSPN contributes’. The definition for iSPN substitutes negative feedback.

A separate problem for the disclosed loop refinement of the classic model is posed by the fact that dSPN collaterals invade both BG output nuclei, SNr and GPi, as well as GPe (Levesque and Parent 2005). Quantitative analysis of terminal boutons showed that caudate SPN axons terminate unequally in SNr : GPi, by a ratio of about 3:1, with putamen SPN showing the opposite pattern^16^ (Levesque and Parent 2005). It was essentially this same observation—that a point in the striatum can communicate with both SNr and GPi—that was characterised as a ‘split-circuit’ in an earlier open-loop formulation for BG circuitry (hinging on the additional premise that SNr and GPi target separate, non-overlapping zones of frontal cortex) (Joel and Weiner 1994). Even if only a minority of striatal output is diverted into the split-circuit, it would introduce a new realm of functionality to the BG loop—one that is absent from mainstream computational models, which may acknowledge other forms of open-loop architecture but uniformly posit a closed loop for cortical re-entry in regard to a specific motor program (Schroll and Hamker 2013).

This question is best scrutinised with reference to the BG circuits formed by areas M1 and SMA, which have been examined most intensively. Kaneda et al. (2002) used orthodromic activation to map the respective ventrolateral (M1) and dorsomedial (SMA) target zones within the putamen, whose input/output connections were then examined using dual tracers; the connections of an intermediate zone where SPNs were jointly activated, by both M1 and SMA, were also studied. The general observation was that the three zones in the putamen formed parallel output to the GPi, with minor overlap, but produced far more convergent, almost fully overlapping projections to SNr (albeit with a very minor contribution from M1). The summary cortico-BG circuit diagram for this study echoes the earlier ‘split-circuit’ scheme of Joel and Weiner (1994). It is posited as an open-loop scheme because the onward nigro-thalamocortical pathways target broad expanses of prefrontal cortex, but exclude areas SMA and M1 (Middleton and Strick 2002; Akkal et al. 2007).

Does the documented presence of a post-striatal ‘split-circuit’ necessarily violate the closed-loop formulation for the re-entrant BG pathway? Perhaps not: although the tracer injections were accurately placed into the SMA zone of the putamen, they revealed widespread sources of cortical input, including premotor areas F2, F4 and F5 as well as F3 (SMA) and F6 (pre-SMA) (Kaneda et al. 2002). Unlike SMA, areas such as F2 are known to receive nigro-thalamocortical input in addition to pallido-thalamocortical input. Indeed, transneuronal retrograde study has shown that rostral and caudal parts of F2 receive input from separate BG output channels, each of which is centred in GPi, but also has an extension within SNr (Saga et al. 2011). The alternative account is, therefore, (a) that BG output channels may have a ‘fuzzy’ boundary between GPi and SNr, such that an individual dSPN sending axon collaterals to each nucleus may still contribute to no more than one output channel; (b) that any large injection of tracer in striatum is likely to encompass SPNs with input from several distinct cortical areas; here, this could include SPNs with operative input from area F2 that establish loops utilising SNr. Thus, the existence of post-striatal open-loop circuitry remains a possibility, but has yet to be demonstrated beyond reasonable doubt.

### Multiple operative input?

There is one final taxing question, which concerns the possibility of multiple operative inputs to a single SPN. Motor areas, for example, can certainly converge upon common striatal territory, but they may observe some restrictions. So much may be inferred from Box 2, documenting examples of the YVH principle. All known exceptions to the YVH principle involve failures of corticostriatal convergence between motor areas that do exchange corticocortical connections. This pattern might be construed as a strategy to segregate operative inputs to the striatum. Where multiple motor inputs do converge on a single SPN one, but only one, should be operative and the remainder contextual—or so the theorising runs, up to this point. Take, for example, an SPN receiving input from both M1 and SMA (Kaneda et al. 2002): does it contribute to both M1 and SMA output channels, or just one? If it does contribute to both output channels, it is receiving dual operative inputs, by definition. The pattern of striatopallidal terminals in GPi (Kaneda et al. 2002) does not resolve the question either way, as both the single and dual operative input models would predict overlap, within GPi, of projections from the M1 (or SMA) striatal zone with the M1/SMA convergent zone, exactly as observed. It might require the application of recently developed dual transneuronal retrograde technology (Ohara et al. 2009) (i.e. capable of identifying a SPN double-labelled with tracers transported from M1 and SMA) to settle the issue conclusively.

## Summary: definitions and conclusions

An anatomical account and functional theory of trans-striatal signal processing is developed in accord with the standard interpretation that the BG play a role in action selection, and as a refinement of the classic model of direct and indirect cortico-BG circuits, originating from twin classes of the striatal spiny projection neuron (dSPN and iSPN). New terminology is proposed within this conceptual framework.

### Striatal input/output architecture

The sources of cortical input to the striatum are broader in origin than the zone of cortex in receipt of BG loop feedback. The latter is currently known (in the primate brain) to comprise frontal cortex plus post-Rolandic areas TE and AIP. This topologically discontinuous territory might be expanded by future discoveries, and is herein referred to as ‘BG-recipient’ cortex.

By definition, non-BG-recipient cortex is external to any form of cortico-BG loop. The term *‘*disclosed loop’ is introduced to summarise cortico-BG circuitry that has both open and closed characteristics at the corticostriatal input stage. Closed- and open-loop corticostriatal afferents are, respectively, termed ‘operative’ and ‘contextual’. The definition is dependent on the focal nature of the re-entrant sector of the BG loop, also known as BG output channels: operative input to a dSPN is the afference it receives from the specific zone of cortex that receives positive feedback from the BG output channel(s) to which that dSPN contributes. The definition for iSPN substitutes negative feedback.

The disclosed loop thesis supposes that the receipt of operative signals is integral to SPN function, as this establishes the bid for action selection; hence, as a corollary, that all SPN situated in all parts of the striatum must receive (operative) input from some part or other of BG-recipient cortex—an anatomical premise that accords with existing evidence, but is yet to be universally demonstrated.

The origin of contextual afferents to a striatal locus is distributed across both BG-recipient and non-BG-recipient cortex, and mirrors the transcortical network harnessed by the source of the operative input to that locus, thereby capturing a similar range of contingencies relevant to determining action. This pattern is summarised by the YVH^17^ principle, here recast to the effect that ‘convergent striatal connections always derive from areas that are cortically connected’. Absence of convergence has been noted for certain pairs of reciprocally connected motor areas, which could be interpreted as a tendency to segregate operative inputs from one another.

### Differential signalling by dSPN and iSPN

By virtue of sparse corticostriatal connectivity, SPNs recognise idiosyncratic, distributed contextual states. The particular PDS^18^ model of SPN biophysics lends itself to the proposal that contextual synapses upon SPN distal dendrites regulate the frequency of SPN Up states, whilst operative synapses upon proximal dendrites control the precise firing pattern. Operative and contextual synapses both undergo plastic changes in efficacy, contingent upon dopaminergic reward. Plasticity is opposite in direction between dSPN and iSPN, due to their different receptors (D1R vs. D2R).

A single operative afferent terminal contacts both classes of SPN, eliciting co-activation of dSPN and iSPN pertaining to the same action: dSPNs signal positive action values and iSPNs negative action values. In context-free (or context-neutral) paradigms the action value represents the plastic state of the operative synapse; otherwise, the momentary balance of positive and negative action values is contingent on the contextual state, leading to action selection or cancellation.

The two classes of corticostriatal afferents, IT and PT, subtending a planning–execution (efference copy) dimension of cortical signalling, are each capable of serving as context or operative input upon dSPN and iSPN alike. Crossed IT afferents, for instance, may convey the motor context of contraversive action planning. PT afferents could play a specific role in action termination, given their preferential contacts upon iSPN.

### Post-striatal loop architecture

The so-called ‘bridging collaterals’, from dSPN to GPe, may enact a form of mutual suppression between rival bids for action selection. dSPN collateral branching to both BG output nuclei (GPi and SNr) might constitute a ‘split-circuit’ (amounting to open-loop architecture in mid-loop, as well as at the corticostriatal stage)—a functional arrangement that is not presaged by computational BG network models. The alternative is that a single BG output channel is diffusely constituted between SNr and GPi.

Some SPNs receive twin motor input (e.g. M1 and SMA). These would be classified as dual operative inputs if the SPN were to contribute to both the respective M1 and SMA output channels. Such fine details of BG circuit organisation have yet to be ascertained.

## Box 1: The main cortico-BG loop plus subcortical sub-loops

There are multiple sub-loops within BG circuitry whose function is little known, and little modelled. (1) The BG output channels from GPi/SNr that contact specific thalamic relay nuclei VL (ventral lateral) and VA (ventral anterior) exhibit a short-circuit directly back to striatum (McFarland and Haber 2000); the medial dorsal nucleus (MD) may be a lesser contributor. This projection is formed by collaterals of thalamocortical afferents, and thereby acts to replicate the positive/negative feedback effects of the direct/indirect pathways, although (2) the corticothalamic projection contacts these same thalamocortical relay cells, hence it is the state of the cortico-thalamocortical oscillation that is influenced by BG output channels and reported directly to striatum. (3) Branches from the BG output channels contact the caudal intralaminar nuclei of the thalamus, CM and Pf (centromedian and parafascicular) that also issue an excitatory thalamostriatal projection. This targets SPNs (and cholinergic interneurons) within the matrix compartment of the striatum, but unlike the projection from VL/VA, terminates preferentially upon dendritic shafts rather than the spines of SPNs. The topographic organisation of this sub-loop retains the same sensorimotor, cognitive and limbic subdivision apparent in the main loop (Sadikot and Rymar 2009). (4) The CM/Pf also sends minor outputs to several other BG nuclei: GPe, GPi, SNr and STN (not illustrated) (Tande et al. 2006). (5) The CM/Pf receives multiple inputs from brainstem structures that also receive direct input from GPi/SNr, such as the SC (superior colliculus) and pedunculopontine nucleus (not illustrated) (Sadikot and Rymar 2009). (6) A subpopulation of about 20–30 % of GPe neurons has no onward output to STN and GPi/SNr, but projects back to the striatum where it terminates non-selectively upon SPNs and striatal interneurons (Kita et al. 1999; Sato et al. 2000a; Mallet et al. 2012). The GPe receives axon collaterals from dSPNs in addition to the canonical projection from iSPNs (Levesque and Parent 2005), so both SPN classes could participate directly in this striato-pallidostriatal loop. (7) The reciprocal loop between GPe and STN is, by contrast, a fixture in several BG network models (see main text). (8) Reward circuitry—the projection to SNc originating from striatal striosomes and the diffusely organised dopaminergic back connection—has received extensive theoretical attention (e.g. Montague et al. 1996; Morita et al. 2012; Collins and Frank 2014); lighter SNc innervation of other BG nuclei (GPe, GPi and SNr, not illustrated) has not (Schroll and Hamker 2013).

## Box 2: Testing the ‘YVH’ principle

Yeterian and Van Hoesen (1978) phrased it thus: “areas of cerebral cortex having reciprocal corticocortical connections, while having unique overall patterns of projection to the caudate nucleus, project, in part, to one and the same region of the nucleus”. Although they specifically cite the caudate nucleus, the Table below tests the principle over the whole striatum, including the putamen.

**Table:**

	Corticostriatal convergence					
	YES		Ref	NO		Ref
Cortico-cortically inter-connected						
YES	S1 (area 3a)	S1 (area 3b)	1	PMv	M1	5
	S1 (area 3a)	S1 (area 1)	1	PMd (F2)	M1	5
	S1 (area 3b)	S1 (area 1)	1	PMv	PMd (F2)	5
	M1	S1	2, 3	CMAc (24d)	SMA (F3)	11
	M1	SMA (F3)	4, 5	CMAc (24d)	CMAr (24c)	11
	Cntrl. M1	M1 (face)	2			
	Cntrl M1	SMA (F3)	6			
	Cntrl SMA	SMA (F3)	10			
	Cntrl A46	A46	10			
	PMd (F2)	SMA (F3)	5			
	PMv	SMA (F3)	5			
	M1	CMAc (24d)	11			
	Pre-SMA (F6)	CMAr (24c)	11			
	FEF	SEF	7			
	PPC (7A)	DLPFC (A46)	8			
	OFC	DLPFC (A46)	8			
	A46	PMd (F7)	12			
	A9	PMd (F7)	12			
	A9	SEF	12			
NO				Cntrl M1	M1 (limb)	2
				FEF	Pre-SMA (F6)	7
				SEF	PMv (F5)	7
				ACC	DLPFC (A46)	8
				SMA(F3)	Pre-SMA (F6)	9
				CMAc (24d)	Pre-SMA (F6)	11
				CMAr (24c)	M1	11
				CMAr (24c)	SMA (F3)	11

*cntrl* contralateral, *PMd and PMv* dorsal and ventral premotor cortex, *SMA* supplementary motor area, *CMAc and CMAr* caudal and rostral cingulate motor areas, *FEF and SEF* frontal and supplementary eye fields, *PPC* posterior parietal cortex, *DLPFC* dorsolateral prefrontal cortex, *OFC* orbitofrontal cortex, *ACC* anterior cingulate cortex

The table lists only the firm conclusions reached by dual anterograde tracer studies of corticostriatal projections to the striatal matrix, in primates. Single tracer studies suggest likely additions—both to the YES/YES box (e.g. convergent projections from visual areas V4, TEO and TE), and to the NO/NO box (e.g. lack of striatal convergence between M1 and pre-SMA (F6) that also lack a direct interconnection; Luppino et al. 1993)—but these have yet to be verified. Note that the examples citing somato-motor areas all specify convergence between equivalent somatotopic loci; there is one example of somatotopic variation, concerning bilateral integration of M1, where midline face and trunk regions have greater interhemispheric connectivity and striatal convergence than non-midline regions (Flaherty and Graybiel 1993b).

There is an arbitrary element in assigning cases according to a simple YES/NO classification. Striatal coincidence tends to parallel cortical network overlap, but quantitative evaluation is rarely available. For the entries pertaining to cingulate motor areas, the criterion for striatal convergence is set at >5 % of the total (i.e. dual) terminal volume in the striatum (Takada et al. 2001); similarly, cingulate cortical areas were classified as ‘connected’ to another area if >5 % of total labelled cortical neurons obtained from the area in question (Hatanaka et al. 2003). In general, the cortical networks formed by caudal vs. rostral motor areas (i.e. M1, SMA/F3, CMAc/24d v. pre-SMA/F6, CMAr/24c) are largely separate (Luppino et al. 1993) and their striatal fields do not overlap. Even so there are several entries in the ‘aberrant’ (corticocortical YES/corticostriatal NO) box. It should be noted that in all examples, the evidence is limited: typically there is only a single case to show the absence of striatal convergence, and no documentation of transcortical connectivity between pairs of injection sites; these entries therefore have a provisional flavour.

By contrast, there is a striking absence of entries in the lower-left box (corticocortical NO/corticostriatal YES)—or in other words, a dearth of evidence for corticostriatal convergence between non-connected cortical areas. This suggests the YVH principle could be rephrased as follows to better comply with existing connectional data: *areas of cerebral cortex having convergent striatal projections (i.e. coincident patches) are reciprocally interconnected, and/or have significantly overlapping networks of cortical connections*. The rephrased version can tolerate examples of reciprocally connected areas failing to make convergent striatal connections. This should help to refine the functional criteria ultimately determining corticostriatal convergence.

Key: 1, Flaherty and Graybiel (1991); 2, Flaherty and Graybiel (1993b); 3, Flaherty and Graybiel (1995); 4, Inase et al. (1996); 5, Takada et al. (1998b); 6, Takada et al. (1998a); 7, Parthasarathy et al. (1992); 8, Selemon and Goldman-Rakic (1985); 9, Inase et al. (1999); 10, McGuire et al. (1991); 11, Takada et al. (2001); 12, Calzavara et al. (2007).

## Abbreviations

BG: Basal ganglia
GPe: External globus pallidus
GPi: Internal globus pallidus
SNr: Substantia nigra pars reticulata
SNc: Substantia nigra pars compacta
SPN: Striatal (spiny) projection neuron
STN: Subthalamic nucleus
PT: Corticostriatal neuron with axon passing into the pyramidal tract
IT: Corticostriatal neuron with axon remaining intratelencephalic

## Motor areas of cortex

M1: Primary motor cortex
PMv: Ventral premotor cortex
PMd: Dorsal premotor cortex
SMA: Supplementary motor cortex
FEF: Frontal eye field
SEF: Supplementary eye field
F2r: Rostral premotor area F2
F2c: Caudal premotor area F2
CMAc: Caudal cingulate motor area
CMAr: Rostral cingulate motor area

## Other areas of cortex

LIP: Lateral intraparietal area
AIP: Anterior intraparietal area

## Motor thalamic nuclei

VA: Ventral anterior
VL: Ventral lateral
MD: Medial dorsal

## Footnotes

1 This is particularly the case for optogenetic and other applications in transgenic mice.

2 A few select models will illustrate certain computational principles; many other variants exist.

3 This relates to the distinction between ‘PT’ and ‘IT’ corticostriatal neurons, shown later in Table 1.

4 The absence of input from V1 (striate cortex) is fortunate, inasmuch as it obviates reference to a ‘striatostriatal’ projection.

5 The lack (until recently) of a ‘dual label’ viral technology prevented use of a dual cortical injection site strategy to infer topography within a single case.

6 For clarity of reference, the term ‘overlap’ will be used to refer exclusively to fields of patches, i.e. all the territory enclosed within a boundary described by the outermost patches; ‘coincidence’ will refer to precise (or partial) superimposition of individual patches, as demonstrated by a double-labelling technique.

7 The connectivity matrix of Markov et al (2014) reports retrograde tracer connections amongst 91 areas; 7A and 46d each connect with 59 other areas, 45 of which are mutual; hence, ‘network overlap’ = 45/59 = 76 %.

8 MR diffusion tractography is incapable of measuring the direction of a connection—the cortex to striatum direction has to be inferred from homology to the monkey.

9 i.e. cortico-striato-nigro-thalamocortical or cortico-striato-pallido-thalamocortical.

10 This is the outcome when the cortex from both hemispheres was subdivided into an arbitrary total of 7 networks; the seventh subdivision, an occipital visual network, had no representation at all within the striatal parcellation.

11 The immunohistochemical techniques in this study were not capable of identifying D1R and D2R in the same tissue (in addition to the retrograde tracer) so co-expression of D1R and D2R was not directly observed, but statistically inferred.

12 This property was documented for both dSPN and iSPN, as identified in BAC transgenic mice.

13 It bears repetition that this is entirely conjectural. There is no direct anatomical evidence for differential termination of PT or IT corticostriatal afferents within the SPN dendritic field, and the PDS model of SPN biophysics itself requires further experimental validation.

14 Even if tonic dopaminergic levels only affect iSPN activity through the more sensitive D2 receptor (Tritsch and Sabatini 2012), this can still regulate the *relative* activity of dSPN and iSPN – in a way that might be mimicked by selective optogenetic stimulation of either cell class.

15 Cazorla et al (2014) found that selective enhancement of the excitability of iSPNs triggered plastic expansion of dSPN collaterals in GPe, but not in SNr; by contrast, selective enhancement of the excitability of dSPNs triggered plastic expansion of dSPN collaterals in SNr, but not GPe.

16 The precise figures cited (for putamen only) were SNr = 17 %, GPi = 55 % and GPe = 28 %.

17 Yeterian and Van Hoesen (1978).

18 Plotkin et al. (2011).
